# Guanine nucleotide binding to the Bateman domain mediates the allosteric inhibition of eukaryotic IMP dehydrogenases

**DOI:** 10.1038/ncomms9923

**Published:** 2015-11-12

**Authors:** Rubén M. Buey, Rodrigo Ledesma-Amaro, Adrián Velázquez-Campoy, Mónica Balsera, Mónica Chagoyen, José M. de Pereda, José L. Revuelta

**Affiliations:** 1 Metabolic Engineering Group, Dpto. Microbiología y Genética. Universidad de Salamanca, Campus Miguel de Unamuno, Edificio Departamental, Salamanca 37007, Spain; 2 Institute of Biocomputation and Physics of Complex Systems (BIFI), Joint Unit IQFR-CSIC-BIFI, Universidad de Zaragoza, C/Mariano Esquillor, Zaragoza 50018, Spain; 3 Department of Biochemistry and Molecular and Cell Biology, University of Zaragoza, Zaragoza 50009, Spain; 4 Instituto de Investigaciones Sanitarias de Aragón (IIS-A), Zaragoza 50009, Spain; 5 Fundación ARAID, Government of Aragón, Zaragoza 50018, Spain; 6 Department Abiotic Stress, Instituto de Recursos Naturales y Agrobiología (IRNASA-CSIC), Cordel de Merinas 40-52, Salamanca 37008, Spain; 7 Computational Systems Biology Group, Centro Nacional de Biotecnología (CNB-CSIC), Darwin 3, Madrid 28049, Spain; 8 Instituto de Biología Molecular y Celular del Cáncer (CSIC-Universidad de Salamanca), Campus Miguel de Unamuno, Salamanca 37007, Spain

## Abstract

Inosine-5′-monophosphate dehydrogenase (IMPDH) plays key roles in purine nucleotide metabolism and cell proliferation. Although IMPDH is a widely studied therapeutic target, there is limited information about its physiological regulation. Using *Ashbya gossypii* as a model, we describe the molecular mechanism and the structural basis for the allosteric regulation of IMPDH by guanine nucleotides. We report that GTP and GDP bind to the regulatory Bateman domain, inducing octamers with compromised catalytic activity. Our data suggest that eukaryotic and prokaryotic IMPDHs might have developed different regulatory mechanisms, with GTP/GDP inhibiting only eukaryotic IMPDHs. Interestingly, mutations associated with human retinopathies map into the guanine nucleotide-binding sites including a previously undescribed non-canonical site and disrupt allosteric inhibition. Together, our results shed light on the mechanisms of the allosteric regulation of enzymes mediated by Bateman domains and provide a molecular basis for certain retinopathies, opening the door to new therapeutic approaches.

## Abstract (web summary)

IMP dehydrogenase (IMPDH) plays essential roles in purine metabolism and cell proliferation. Here Buey *et al*. describe a guanine nucleotides regulated molecular mechanism for allosteric communication between the regulatory and catalytic domains of IMPDH.

## Graphical abstract

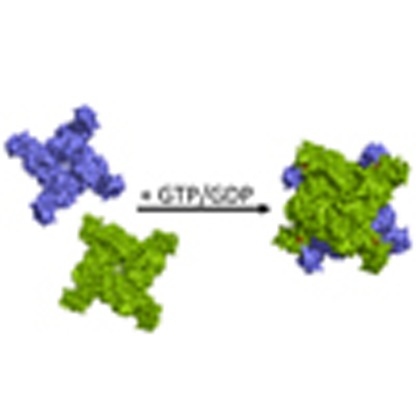

Purine nucleotides are essential molecules for the cell. They not only constitute the building blocks of nucleic acids but also play central roles in metabolism, become incorporated into enzyme cofactors, represent the energy source for translation and microtubule polymerization, and are involved in signal transduction, angiogenesis^1^ and axon guidance^2^.

In general, cells synthesize purine nucleotides in two different ways: in the *de novo* pathways, the purine ring system is assembled in a step-wise manner from biosynthetic precursors of carbohydrate and amino acid metabolism. In contrast, the *salvage* pathways recycle preformed nucleobases, nucleosides and nucleotides. Both biosynthetic pathways are very tightly regulated, to maintain an appropriate balance between adenine and guanine nucleotide pools, as well as an optimal energy charge along the different stages of the cell cycle.

Within the *de novo* purine biosynthetic pathway, inosine-5′-monophosphate (IMP) is the first molecule in the pathway to have a completely formed purine ring system and is the common precursor at the branch point of the adenine and guanine nucleotide *de novo* pathways. The enzyme IMP dehydrogenase (IMPDH, EC 1.1.1.205) catalyses the oxidative reaction of IMP to xanthosine 5′-monophosphate (XMP), which is subsequently converted to guanosine-5′-monophosphate (GMP) in a reaction catalysed by the enzyme GMP synthase.

The reaction catalysed by the IMPDH represents the rate-limiting step in guanine nucleotide biosynthesis and hence IMPDH is an essential enzyme that controls the cellular pool of guanine nucleotides, playing crucial roles in functions such as the immune response^3^ or cell proliferation^4^. Accordingly, the therapeutic potential of IMPDH has been explored intensively in the last two decades, which has resulted in a diverse group of drugs with antitumour, antiviral, antiparasitic, antibacterial and immune-suppressive activities, including mycophenolic acid (CellCept), mizoribine (Bredinin) and ribavirin (Virazole and Rebetol), which are at present widely used in clinical chemotherapy^5^.

In addition to its therapeutic potential, the manipulation of the *IMPDH* gene can be used to modulate the metabolic flux through the guanine nucleotide *de novo* biosynthetic pathway with a view to improving the production of metabolites of industrial interest whose direct precursor is GTP. For instance, in the industrial filamentous fungus *A. gossypii*, a natural overproducer of riboflavin (vitamin B2), the manipulation of the *IMPDH* gene—by means of metabolic engineering approaches—significantly increased the production of riboflavin^6^.

IMPDH forms tetramers in solution, each monomer consisting of a catalytic and a regulatory domain. The catalytic domain is a (β/α)_8_ barrel, which represents the archetypal triose-phosphate isomerase fold (TIM barrel^7^). A special feature of IMPDHs is the presence of a twisted β-sheet that projects outwards from the carboxy-terminal face of the TIM barrel. This structure, called the ‘finger domain', is present in all known IMPDHs, although its precise function remains unknown. The regulatory part, ∼120 amino acids long, is inserted within a loop of the catalytic domain and is composed of two repeats of the cystathionine β-synthase (CBS) domain, constituting a CBS pair or Bateman domain^8^.

Bateman domains are also present in a variety of proteins such as voltage-gated chloride channels, AMP-activated protein kinase and CBS, where they regulate protein function in response to the binding of adenosyl molecules^9^,^10^,^11^,^12^. The importance of Bateman domains is underlined by the fact that mutations in them cause a variety of human hereditary diseases, including the Wolff–Parkinson–White syndrome, congenital myotonia, homocystinuria and so on^9^. In IMPDH, missense mutations in the Bateman domain are linked to Leber congenital amaurosis (LCA) and retinitis pigmentosa (RP)^13^. The Bateman domain has little impact on the catalytic activity and inhibitor binding, as it has been shown for several IMPDHs^6^,^14^,^15^,^16^, but has been associated with single-stranded DNA binding^17^,^18^ and in allosteric regulation by ATP^16^. Nonetheless, there is limited knowledge regarding the molecular mechanisms responsible for the communication between the Bateman domain and the catalytic core of IMPDH. Moreover, contradictory information about the physiological regulation of human IMPDHs has been reported. For instance, GTP has been reported to bind to purified human IMPDH isoform 2 (HsIMPDH2) at physiological concentrations^19^ but this finding has not been corroborated by other authors^20^. Similarly, ATP has been described to bind to HsIMPDH2 and to increase its activity^9^ but, again, this finding has not been confirmed by other authors^16^,^17^,^20^.

In this work we have used a multidisciplinary approach to study the physiological regulation of IMPDH by purine nucleotides, using *A. gossypii* as a model. We show that GTP and GDP induce the association of the Bateman domains of AgIMPDH to form octamers. In these octamers, the interaction of the finger domains decreases the apparent affinity for the substrate IMP, reducing the catalytic activity. We also report the first high-resolution structure of a Bateman domain bound to guanine nucleotides and describe the structural determinants that dictate adenine or guanine nucleotide binding. Moreover, our data suggest that eukaryotic and prokaryotic IMPDHs might have adopted different mechanisms of allosteric regulation, with GTP and GDP inhibiting only eukaryotic IMPDHs. Interestingly, missense mutations in the Bateman domain linked to human RP and LCA^13^ map into the guanine nucleotide-binding sites, including a previously undescribed non-canonical site. Taken together, our results not only shed light on the mechanisms of the allosteric regulation of enzymes mediated by Bateman domains but also provide a molecular basis for human retinopathies and open the door to new potential therapeutic strategies.

## Results

### AgIMPDH activity is inhibited by guanine nucleotides

**Figure 1:**
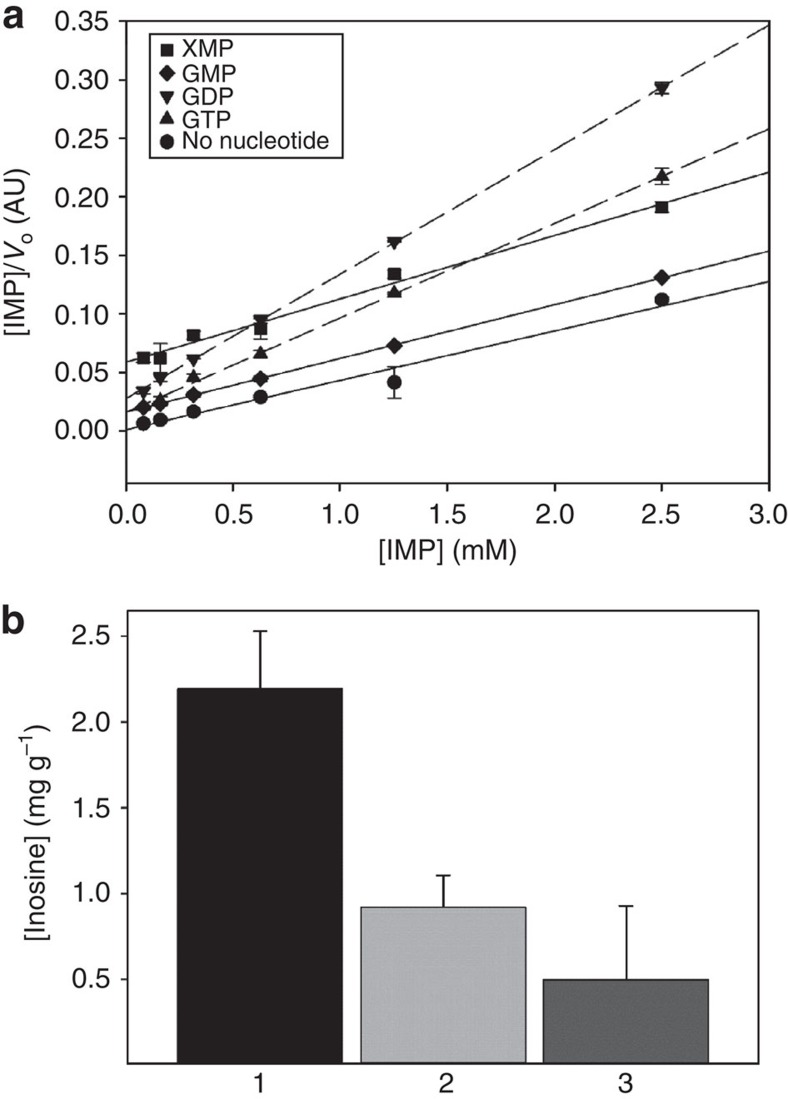
GTP and GDP allosterically inhibit AgIMPDH. (**a**) Analysis of the enzyme kinetics *in vitro* data using the linear Hanes-Woolf plot. Initial velocities (*V*_o_) were determined by fitting the time-course data to the Michaelis–Menten equation. Reactions contained 20 μg ml^−1^ AgIMPDH, 0.5 mM NAD^+^ and variable concentrations of IMP. Reaction buffer (100 mM Tris-HCl, 100 mM KCl, 2 mM DTT pH 8.0) did not contain Mg^+2^. (**b**) Milligrams of inosine excreted per gram of mycelium from different strains of *A. gossypii* (1: WT, 2: *ΔBateman* and 3: *P*_*GPD*_*-ΔBateman*) after 3 days of culture in MA2-rich medium at 28 °C with orbital shaking. Experiments were performed in triplicate. Error bars represent s.e.

AgIMPDH is a tetramer in solution with optimal activity at pH 8.0 in the presence of 100 mM K^+^ ions; it shows Michaelis–Menten kinetics and is competitively inhibited by both the XMP product and the NAD^+^ substrate^6^. Given the central role of IMPDH activity in the metabolism of purine nucleotides, our aim was to study the effects of adenine and guanine nucleotides on IMPDH catalytic activity *in vitro*. Enzyme kinetic measurements revealed that GMP, guanosine 5′-di- and triphosphate (GDP and GTP, respectively) significantly inhibited AgIMPDH *in vitro* (Fig. 1 and Supplementary Fig. 1). In contrast, the corresponding adenine nucleotides AMP, ADP and ATP did not exert any significant effect (Supplementary Fig. 2A). Analysis of the enzyme kinetic curves revealed that GMP is a competitive inhibitor with respect to IMP, with *K*_i_=600±100 μM, whereas GDP and GTP do not compete either with IMP or with NAD^+^, that is, they are mixed-type (allosteric) inhibitors of AgIMPDH, with *K*_i_=210±40 and 160±40 μM, for GDP and GTP, respectively (Table 1). The differences in the mechanisms of inhibition are clearly visible using linear Hanes–Woolf plots, where lines parallel to the control sample (no inhibitor) indicate competition, in contrast to mixed-type inhibitors that produce lines with different slopes than the control (Fig. 1a).

We further observed that GTP and GDP might bind to the Bateman domain, because the enzymatic activity of a mutant lacking this domain (ΔBateman) was not significantly affected either by GDP or by GTP, in contrast to GMP that inhibited the wild-type and ΔBateman enzymes in a similar manner (Supplementary Fig. 2B). ΔBateman and AgIMPDH displayed similar catalytic efficiencies, corroborating previous reports showing that the Bateman domain of IMPDHs is dispensable for catalytic activity^14^,^15^,^16^. We thus conclude that the Bateman domain of AgIMPDH is dispensable for catalytic activity, but that it is responsible for the allosteric inhibition mediated by both GDP and GTP.

To confirm the significance of AgIMPDH allosteric regulation *in vivo*, we constructed an *A. gossypii* strain expressing an *IMPDH* gene lacking the Bateman domain (*ΔBateman*) and another one where the *ΔBateman* deletion mutant was overexpressed (*P*_*GPD*_*-ΔBateman*). We then quantified the concentrations of inosine excreted to the culture media, as these are directly correlated with the intracellular levels of IMP^21^. Remarkably, deletion of the regulatory domain (*ΔBateman*) significantly reduced excreted inosine levels with respect to the wild type, an effect that was more noticeable when the *ΔBateman* mutant was overexpressed (Fig. 1b). These data suggest that the deletion of the regulatory Bateman domain significantly increases the metabolic flux through the guanine nucleotide pathway as a consequence of the allosteric deregulation of the AgIMPDH enzyme.

### GDP and GTP alter the oligomeric state of AgIMPDH

**Figure 2:**
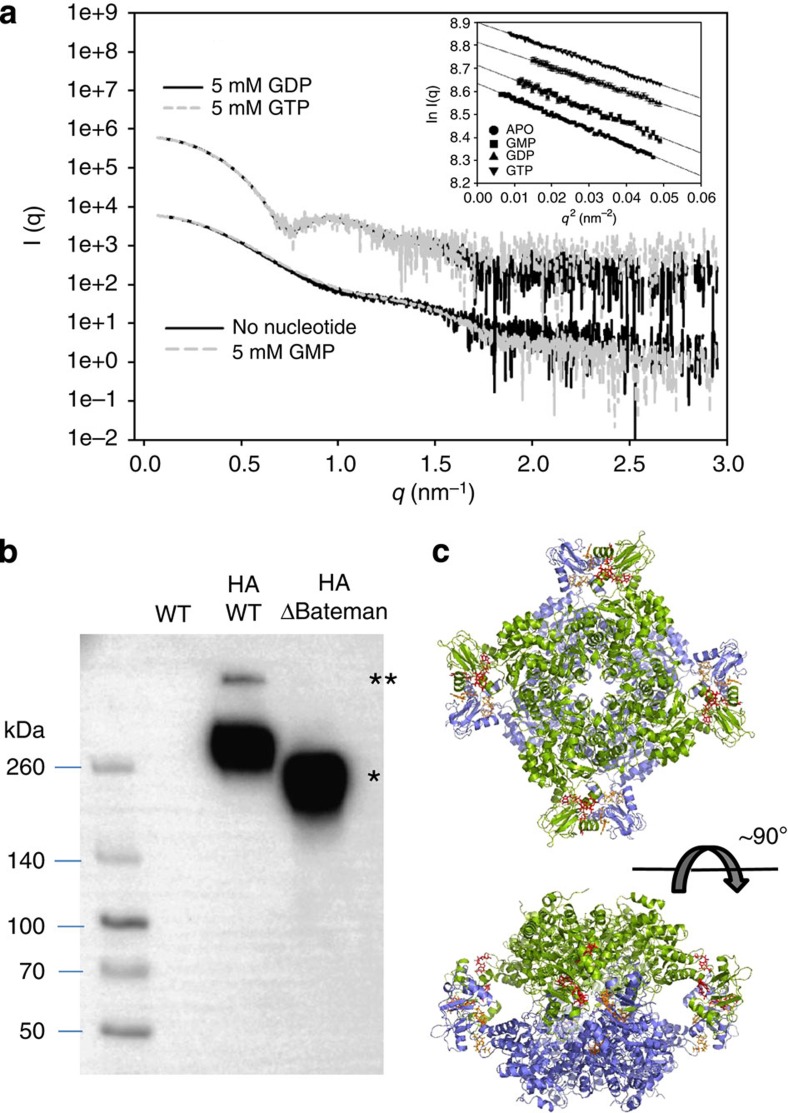
GTP and GDP alter the quaternary structure of AgIMPDH. (**a**) SAXS profiles of AgIMPDH in the presence of different nucleotides. The inset shows the Guinier plots for the different curves. The plots have been conveniently displaced along the *y* axis to facilitate visualization. (**b**) Anti-HA western blotting of cross-linked *A. gossypii* whole-cell extracts. The single and double asterisks show the bands unambiguously attributed to tetramers and octamers, respectively. (**c**) Different views of a cartoon representation of AgIMPDH octamers, obtained in the presence of GDP (red and orange sticks). The two tetramers that pile up tail-to-tail are coloured green and blue.

To further investigate the molecular mechanisms mediating AgIMPDH inhibition by guanine nucleotides, we used small angle X-ray scattering (SAXS) to determine the size and shape of AgIMPDH in solution. The estimated radius of gyration, maximum intra-particle distance and volume of the scattering particle were consistent with a globular protein of about 240 and 180 kDa for AgIMPDH and ΔBateman, respectively (Table 2 and Fig. 2a). GMP induced a slight compaction of the structure (Rg is about 1 Å smaller). By contrast, GTP and GDP induced a pronounced reorganization in AgIMPDH that duplicated the scattering particle's volume (Table 2). Therefore, these changes are compatible with the association of two tetramers to form an octamer. Within the experimental error, identical hydrodynamic parameters were found across the whole concentration range assayed (1.25–20 mg ml^−1^), indicating that AgIMPDH octamerization was not dependent on the concentration of protein but rather on the concentration of nucleotide.

The SAXS data were further supported by chemical cross-linking followed by SDS–polyacrylamide gel electrophoresis experiments that yielded similar results (Supplementary Fig. 3A). Cross-linking reactions at different concentrations of GDP and GTP allowed us to estimate the percentage of tetramers and octamers by band densitometry. Remarkably, an inverse correlation was seen between octamer formation and AgIMPDH activity (Supplementary Fig. 3B). Thus, it may be concluded that the binding of GTP or GDP to the Bateman domain of AgIMPDH mediates its allosteric inhibition by inducing the formation of octamers with compromised catalytic activity.

To test whether we could detect these octamers in cells, we constructed strains where AgIMPDH or ΔBateman proteins were tagged by inserting the haemagglutinin (HA) epitope at the α7–β10 loop, observed to be exposed to the solvent in our crystal structures (see below). Anti-HA western blot analysis of cross-linked whole-cell extracts from these strains featured major bands of AgIMPDH tetramers as well as an additional upper band that is present only in the wild-type HA-tagged protein (Fig. 2b). The upper band corresponded to AgIMPDHs octamers, indicating that these species also exist in cell extracts.

### GTP and GDP force the interaction of the finger domains

To further elucidate the molecular mechanisms underlying the physiological inhibition of IMPDHs, we sought to determine the high-resolution crystallographic structures of AgIMPDH bound to guanine nucleotides. Attempts to crystallize full-length wild-type AgIMPDH in complex with GMP were unsuccessful. However, we obtained crystals of the ΔBateman deletion mutant, in the presence of 10 mM GMP, which diffracted up to 1.25 Å resolution (Table 3). This structure resembled the previously reported structures in complex with IMP and XMP (PDB codes 4XTI and 4XTD, respectively)^6^. GMP adopts a conformation identical to that of the substrate, thus confirming a pure competitive inhibition mechanism (Supplementary Fig. 4). A detailed description of this structure is provided in the Supplementary Note 1.

We were also able to solve the structure of full-length AgIMPDH, co-crystallized with GDP at 2.25 Å resolution. The crystal belongs to the space group P4 (Table 3) with four monomers in the asymmetric unit (AU), each of them located around a quaternary symmetry axis that generates a tetramer. The electron density for the Bateman domain of each of the four monomers in the AU was clearly visible, including three GDP molecules (see below) and the linker regions that connect the Bateman to the catalytic domain. Surprisingly, although it was not added to the crystallization drop, unequivocal electron density for GMP within the active site was observed. GMP is most probably the product of GDP hydrolysis with time in the crystallization conditions. There was also clear electron density for the finger domains, which are composed of a four-stranded twisted β-sheet that projects from the C-terminal face of each monomeric subunit. Between the second and the third strands of this *β*-sheet extends a long loop (residues Arg415–Thr451) containing residues Met417, Gly418 and Gly491, which are in contact with the substrate, as well as a short α-helix (residues Ile410–Gln424) and the mobile catalytic flap (residues Lys425–Val444), not visible in our structure. In addition, the last ten C-terminal residues form a *β*-strand that packs into the *β*-sheet of the finger domain in the contiguous monomer of the tetramer (Supplementary Fig. 5).

Interestingly, two monomers in the AU are related by a binary non-crystallographic symmetry axis, perpendicular to the quaternary symmetry axis. Thus, AgIMPDH octamers are observed within the crystal lattice, assembled as dimers of tetramers that pile up tail-to-tail (Fig. 2c and Supplementary Fig. 6). These octamers are the most abundant species in solution in the presence of millimolar amounts of either GDP or GTP, as demonstrated by the excellent agreement between their theoretical and experimental SAXS profiles (Supplementary Fig. 7A). Remarkably, in our SAXS experiments we found no evidence of the more extended conformation previously reported for *Pseudomonas aeruginosa* (PaIMPDH) in the presence of ATP-Mg (PDB 4DQW). In turn, our more compact octamer resembled that of the apo form of PaIMPDH, resolved at low resolution by the same authors^16^, as well as those deduced from the crystal packing of *Streptococcus pyogenes* (PDB 1ZFJ) and *Bacillus anthracis* (PDB 3TSB)^22^,^23^ (Supplementary Fig. 7B).

**Figure 3:**
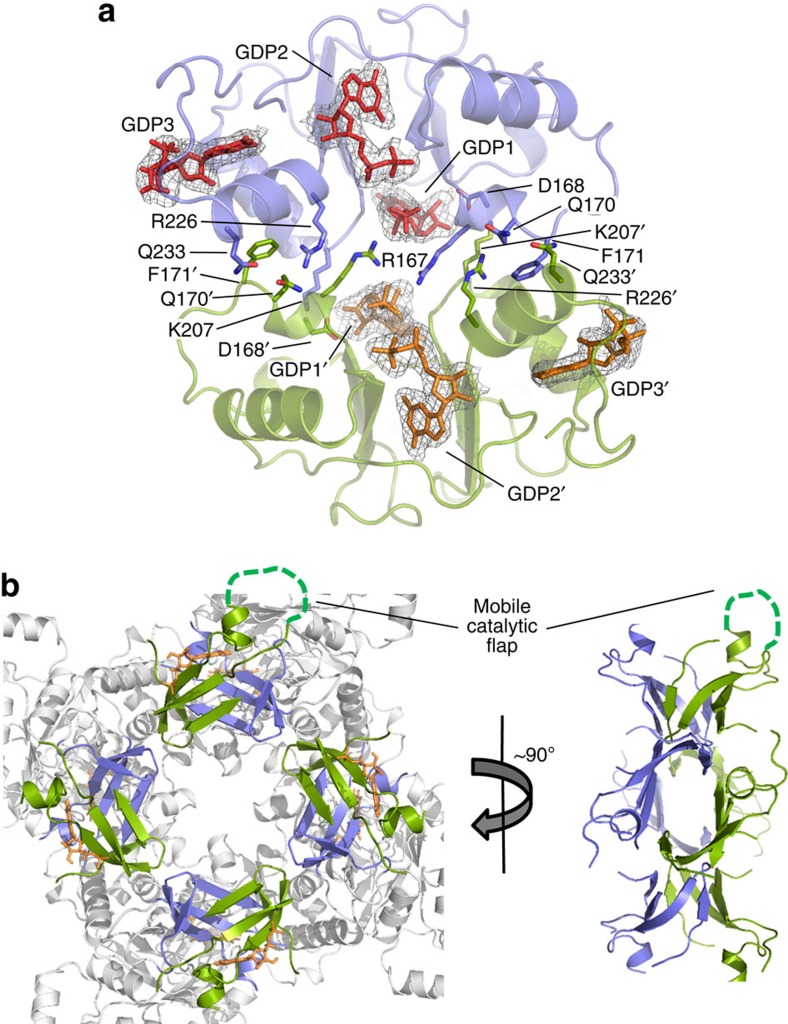
The bipartite interface of the AgIMPDH dimer of tetramers. (**a**) Two Bateman domains from the upper and lower tetramers are shown in green and blue cartoons. GDP-binding protein residues and GDP molecules are shown in sticks. The grey meshes around GDP molecules represent the simulated annealing omit 2mF_o_−DF_c_ electron density maps contoured at the 1*σ* level. (**b**) The finger domains from the upper and lower tetramers are shown in green and blue cartoons around the quaternary (left) and binary symmetry axes (right). GMP bound to the catalytic site is shown with orange sticks in the left panel. The catalytic mobile flap (not visible in our structure) is represented as a discontinuous green line in one of the monomers.

The dimers of the tetramers of AgIMPDH are mainly stabilized by an interface that involves the Bateman domains from the upper and the lower tetramers, which associate to form an antiparallel arrangement that buries a surface of ∼8.800 Å^2^ per monomer. This interface is mostly stabilized by hydrogen bonds between the backbone atoms of residue Arg167 and the side chain of Gln233 in a monomer with the respective side chains of residues Arg226 and Gln170 in the adjacent Bateman domain. Salt bridges between residues Asp168 and Lys207, and the hydrophobic packing of residue Phe171 also contribute to stabilizing the interface. Interestingly, the β-phosphate of a GDP molecule contacts the side chain of Arg167 in the adjacent Bateman domain, further stabilizing the interface (Fig. 3a).

A direct consequence of the interaction of the Bateman domains is that it brings the finger domains of the monomers from both tetramers into contact and arranges them into four pseudo-β-barrels that further associate around the fourfold axis, contributing to the stabilization of the interface by burying a surface of ∼5,000 Å^2^ per monomer (Fig. 3b). The interaction between the finger domains is mostly mediated by hydrogen bonds between residues Tyr403, Phe405 and Asp407 in a single chain with residues Asp522, Lys518 and Lys409 in the adjacent monomer, as well as salt bridges between the side chain of residues Arg406, Arg410 and Glu517 and residues Glu517, Asp 522 and Arg406, respectively (Supplementary Fig. 8A). Notably, similar interactions of the finger domains have been observed within the crystal lattice of the structures of *B. anthracis* (PDB 3TSB^23^) and *S. pyogenes* (PDB 1ZFJ^24^) (Supplementary Fig. 8B), as well as in the low-resolution structure of apo-PaIMPDH^16^. These findings indicate that the interaction of the finger domains might indeed be of relevant functional significance, and that it has been conserved evolutionarily.

The association of AgIMPDH tetramers change neither the structure nor the accessibility of the substrate to the active site significantly, as the tetramers of ΔBateman-GMP perfectly superimpose over AgIMPDH-GDP. Thus, the GTP/GDP-induced decrease in catalytic activity is most probably the consequence of an increase in the rigidity and the subsequent alteration of the internal dynamics of the finger domain, which translates into the loss of apparent affinity for the substrate. The accessibility of the catalytic flap to the active site might also be altered in the octameric context. Indeed, isothermal titration calorimetry (ITC) experiments revealed that the affinity of AgIMPDH for IMP decreases significantly in the presence of GTP and GDP due to an entropic penalty (Table 4).

To further corroborate the relevance of the finger domain in AgIMPDH activity and regulation, we designed two mutations that are predicted to disrupt the structure of the finger domain: (i) a deletion of the last ten C-terminal residues (AgIMPDH-Δ513-522), which form a β-strand that completes the finger domain's β-sheet in the adjacent monomer and (ii) a point mutation of Asp456 to Arg (D456R), a conserved residue that bridges three of the β-strands in the β-sheet to the end of helix α16 in the catalytic domain (Supplementary Fig. 5). Remarkably, both mutants showed a marked reduction in activity (Supplementary Fig. 9), indicating that the finger domain of IMPDHs is not only a key player that mediates the transmission of the allosteric signal from the Bateman to the catalytic domain but it is also essential for catalysis.

### AgIMPDH has three guanine nucleotide-binding sites

An important finding in our work is that the Bateman domain of AgIMPDH specifically binds GTP and GDP. This discovery expands the repertoire of ligands recognized by CBS motifs reported so far^9^,^10^,^11^,^12^ and highlights the crucial role of these domains as sensors of an increasing variety of metabolites and as key regulators of different catalytic activities in response to alterations in the cellular metabolome and/or cell energy levels.

In our structure, we found three GDP molecules bound per Bateman domain: two of them (GDP1 and GDP2) were bound to the canonical nucleotide-binding sites of archetypical CBS motifs, whereas the third one (GDP3) was bound to a previously undescribed site.

**Figure 4:**
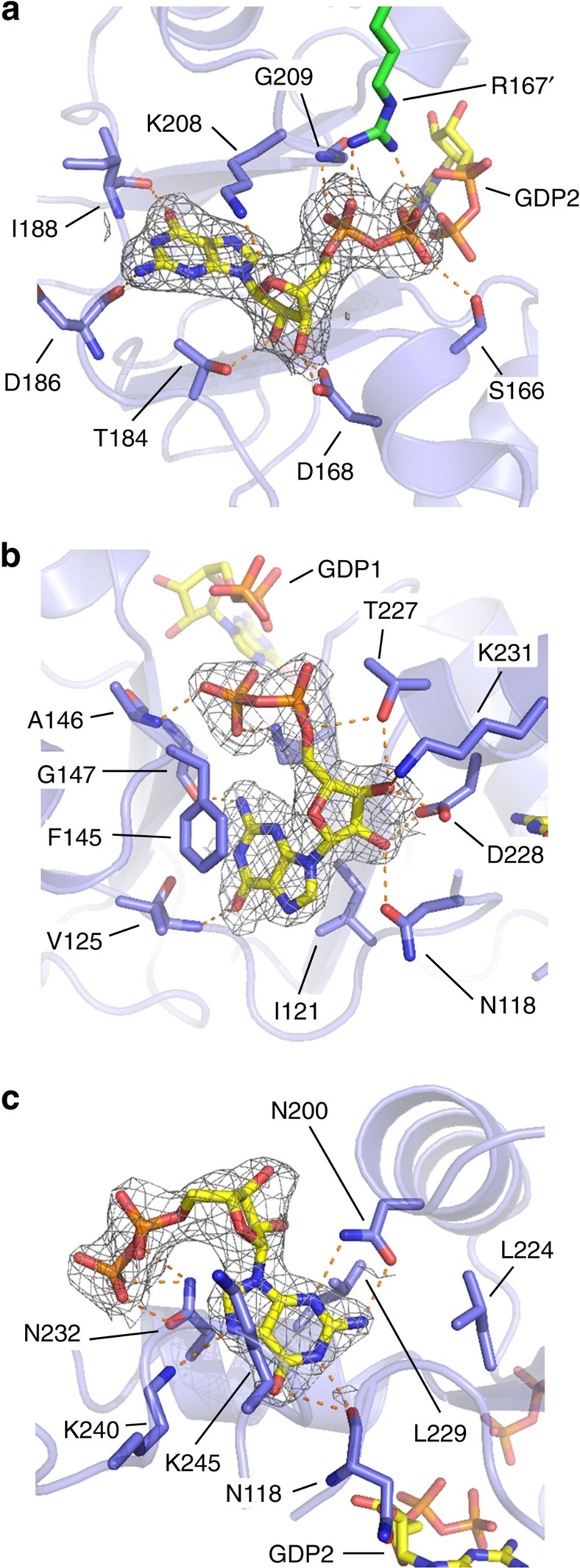
The Bateman domains of AgIMPDH bind guanine nucleotides. Close-up views of the three GDP molecules bound to the Bateman domains of AgIMPDH (**a**) GDP1, (**b**) GDP2 and (**c**) GDP3. AgIMPDH protein is shown in light blue cartoons with key interacting residues and GDP molecules shown in sticks. In **a**, the side chain from an Arg residue from the adjacent monomer (R167') is shown with green sticks. Key interactions are represented by orange dashes. The grey mesh around GDP represents the simulated annealing omit 2mF_o_−DF_c_ electron density map contoured at the 1*σ* level.

GDP1 adopts an extended conformation where the specificity for the guanine ring, sandwiched between the side chains of Lys208 and Thr184, is conferred by hydrogen bonds between atoms N2 and O6 with residues Asp186 and Ile188, respectively. The ribose moiety is stabilized through contacts with the side chains of residues Asp168, Thr184 and Lys208. The phosphates are coordinated by residues Ser166 and Gly209, as well as the side chain of Arg167 from the adjacent tetramer (Fig. 4a).

In contrast to GDP1, GDP2 adopts a compact conformation in which the guanine ring is inverted due to a rotation around the C1–N9 bond that brings N2 and the β-phosphate into contact, both interacting with the backbone of residue Gly147 (Fig. 4b). The guanine ring is sandwiched between the hydrophobic side chains of residues Phe145 and Ile121, and interacts through O6 and N2 atoms with the backbone atoms of residues Val125 and Gly147, respectively. The ribose moiety is stabilized by contacts of the hydroxyl groups with the side chains of residues Asn118, Thr227, Asp228 and Lys231. Finally, the β-phosphate moiety establishes hydrogen bonds with the backbone atoms of residues Gly147 and Ala146, as well as the side chain of Lys210 (Fig. 4b).

GDP3 binds to the cleft formed by the α6 and α7 helices and the α2–α3 loop, which is connected to the canonical binding site in CBS2 by residue Asp 228. The guanine moiety is accommodated into a hydrophobic pocket formed by residues Gly119, Leu196 and Leu224, and sandwiched between the side chains of Leu229 and Lys245. Guanine specificity is conferred by hydrogen bonds with residues Asn118, Asn200 and Lys240 (Fig. 4c). Contrary to the guanine ring, which is positioned identically in all four chains in the AU, the phosphates are not so strongly coordinated and adopt alternate conformations (Supplementary Fig. 10A). Although further studies are needed to decipher the exact significance of this new binding site, the fact that it needs to be intact for GTP/GDP inhibition (see below), that it is well conserved in eukaryotic organisms (Supplementary Fig. 10B) and that three missense mutations linked to human retinopathies^13^,^25^ map into this pocket (see Discussion) indicate that this site indeed has a physiological role. Remarkably, this site is exclusive of eukaryotic enzymes and is not found in bacterial IMPDHs (Supplementary Fig. 10B). To this respect, a previously described non-canonical site (called ‘site E') within the CBS module of the archaeal protein MJ1225 (ref. 26) is structurally unrelated to the site described here (Supplementary Fig. 11).

To corroborate that the specific binding of GTP/GDP to the Bateman domain of AgIMPDH is the event that triggers the allosteric inhibitory signal, we introduced point mutations predicted to disrupt the binding of GDP1 (R167E), GDP2 (F145A), GDP3 (N200K) or the interface between the Bateman domains (R226P). Remarkably, all of these mutations desensitized the enzyme to GTP/GDP inhibition (Supplementary Fig. 12), indicating that the three intact guanine nucleotide-binding sites, as well as the Bateman interface, are necessary for allosteric inhibition. Future experiments are required to dissect the exact contribution of the non-canonical and the canonical sites to the molecular mechanisms that trigger the allosteric signal from the Bateman to the catalytic domain of eukaryotic IMPDHs.

Interestingly, all the three guanine nucleotide-binding sites found in AgIMPDH are well conserved within eukaryotic IMPDHs (Supplementary Fig. 13A). In fact, some of these mutations are found in HsIMPDH1 of patients with either RP or LCA^13^, opening the intriguing possibility of the direct involvement of the GTP/GDP-mediated allosteric regulation of IMPDH in hereditary ocular diseases (see Discussion).

### GTP/GDP do not allosterically inhibit prokaryotic IMPDHs

**Figure 5:**
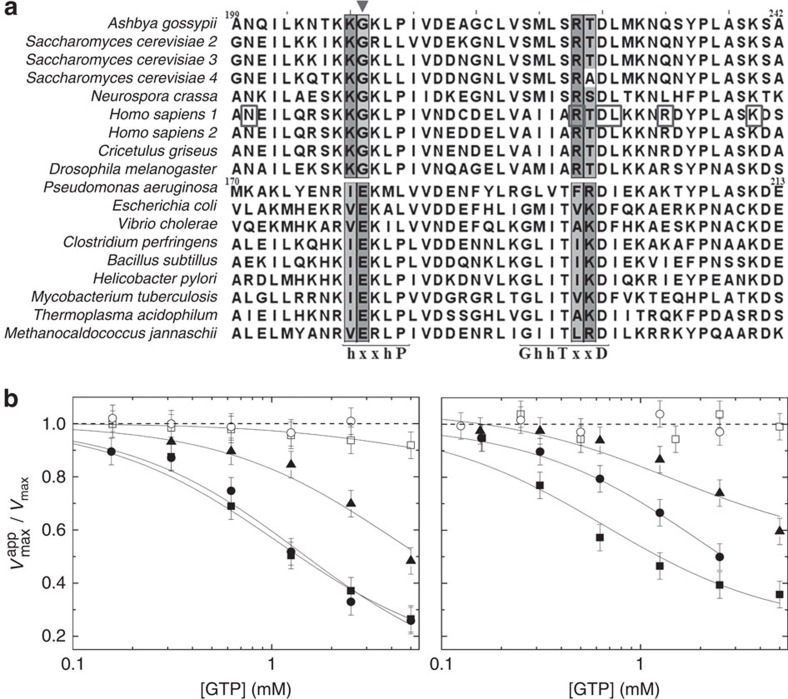
GTP and GDP only inhibit eukaryotic IMPDHs. (**a**) Multiple-sequence alignment of the CBS2 motif within the Bateman domain of selected eukaryotic and prokaryotic organisms. The nucleotide-binding sequence motifs (‘h-x-x-h-P' and ‘G-h-hT-x-x-D')^11^,^12^ are indicated below the sequences. The highest-scoring SDPs are shown in a shaded box. The triangle indicates the glutamate residue within the ‘RIEK' motif of prokaryotic IMPDHs or the glycine within the ‘KKGK' motif that defines eukaryotic IMPDHs. Residues of the human isoform 1 associated with retinopathies are shown in a box. (**b**) Catalytic activity at increasing concentrations of GTP and GDP of AgIMPDH (black squares), HsIMPDH1 (black circles), HsIMPDH2 (black triangles), EcIMPDH (white circles) and BsIMPDH (white squares). The *V*_max_ and *V*_max_^app^ values as a function of inhibitor concentration were determined by fitting the enzyme kinetics data to the Michaelis–Menten equation. Experiments were performed in duplicate. Error bars represents s.e. The continuous lines represent the nonlinear regression fitting analysis employing a mixed inhibition model.

Bacterial IMPDH enzymes have been reported to show biochemical and kinetic characteristics that are different to the mammalian IMPDH enzymes in terms of inhibitor binding and catalytic mechanisms^22^,^24^. In the present study, we extend the scope of this idea by focusing on the different nucleotide interactions within the Bateman domain. Given that PaIMPDH^16^—and some other bacterial enzymes^27^—have been recently reported to be activated allosterically by Mg-ATP in contrast to AgIMPDH, which is inhibited by GTP, we next wished to elucidate the specificity-determinant positions (SDPs^28^) that dictate the binding of adenine or guanine nucleotides. Several SDPs were detected within the Bateman domain (Supplementary Fig. 13A), which unequivocally differentiate IMPDH into two groups: eukaryotic and prokaryotic. Interestingly, the highest-scoring SDPs lay within the archetypal nucleotide-binding sequence motifs ‘*h-x-x-h*-P' and ‘*G-h-x-T-x-x-D*'^11^,^12^ of CBS2 (Fig. 5a), delineating the distinctive allosteric mechanisms of PaIMPDH and AgIMPDH, and a molecular footprint to distinguish those IMPDHs inhibited by GTP/GDP from those which are not. For instance, the requirement of Mg^+2^ ions by ATP to enhance catalytic activity in *P. aeruginosa*^16^ is determined by Glu180 (within what we call the ‘RIEK motif'), which coordinates Mg^+2^ to the γ-phosphate of ATP (Supplementary Fig. 13B). The glutamate residue, strictly conserved in prokaryotic IMPDHs, is replaced by glycine in eukaryotic IMPDHs (Gly209 in AgIMPDH; within what we call the ‘KKGK motif'), which interacts specifically with the α-phosphate of GDP (Supplementary Fig. 13B), which similar to GTP does not require Mg^+2^ to inhibit catalytic activity.

To further corroborate the putative differences in the allosteric regulation between eukaryotic and prokaryotic IMPDHs, we performed *in vitro* tests addressing the effects of purine nucleotides on the activity of the two human isoforms (HsIMPDH1 and HsIMPDH2) and on the IMPDHs from *Escherichia coli* (EcIMPDH) and *Bacillus subtillis* (BsIMPDH). Our data show that GTP and GDP are mixed-type (allosteric) inhibitors of the two human isoforms, but they do not significantly affect the activity of the bacterial enzymes tested (Fig. 5b, Table 1 and Supplementary Figs 14–16). By contrast, GMP is a weak competitive inhibitor of both eukaryotic and prokaryotic IMPDHs (Table 1). In contrast to guanine nucleotides, neither ATP nor Mg-ATP showed a significant effect on the catalytic activity of either eukaryotic or prokaryotic IMPDHs (Supplementary Fig. 17). These results further confirm our hypothesis that only eukaryotic IMPDHs are inhibited allosterically by GTP and GDP, although more experiments would be required to demonstrate that our findings can be generalized to other organisms.

## Discussion

IMPDH is a rate-limiting enzyme in nucleotide biosynthesis that controls the guanine nucleotide pool and hence plays essential roles in cellular metabolism and proliferation. As such, it represents an important therapeutic target that has attracted much attention. Surprisingly, despite its clinical relevance, very limited information is available about the physiological regulation of the enzyme. In this work, we report our finding of a novel molecular mechanism of physiological regulation of eukaryotic IMPDHs. Our data challenge the *in vivo* significance of the classical competitive feedback inhibition of IMPDH by GMP, because the concentrations needed for such inhibition to occur are at least one order of magnitude larger than the expected intracellular concentrations (60±40 μM^29^). Instead, we demonstrate that GTP and GDP are much stronger mixed-type (allosteric) inhibitors of eukaryotic IMPDHs (*A. gossypii* and human), with *K*_i_ values in the range of their expected intracellular concentrations (500±200 and 160±50 μM, respectively^29^).

In contrast to other CBS-containing enzymes^11^,^12^, the binding of GTP/GDP to the Bateman domain of AgIMPDH does not induce significant conformational changes that are further transmitted to the catalytic domain but instead results in the association of the Bateman domains to arrange the protein as a dimer of tetramers with significantly reduced catalytic activity. The octameric arrangement of AgIMPDH not only provides thermodynamic stability for the assembly but also a new spectrum of collective motions and an efficient means of allosteric communication that modulate substrate binding and enzymatic activity. Specifically, the association of the tetramers forces the interaction of the ‘finger domains', evolutionarily conserved structures exclusive to IMPDHs, which we demonstrate to be essential for catalytic activity. The interaction of the finger domains alters substrate binding, presumably due to alterations in protein flexibility around the active site, as suggested by the large entropic penalty measured for the binding of IMP to AgIMPDH octamers. Remarkably, similar interactions have been reported for IMPDHs from other organisms, indicating that the dimerization of tetramers is a conserved property of IMPDHs^16^. In this regard, Labesse *et al*.^16^ have recently reported residual enzymatic activity for wild-type PaIMPDH octamers (*K*_0.5_=1800±100 μM). Interestingly, the finger domains of this enzyme interact in a similar way than our inhibited GDP complex structure. However, when the finger domains of PaIMPDH do no longer interact, that is, on Mg-ATP activation or in a mutant enzyme (PaIMPDH-ΔCBS) with compromised octamer formation, PaIMPDH shows much higher activity (*K*_0.5_=36±4 and 34±2 μM, for PaIMPDH-MgATP and the PaIMPDH-ΔCBS mutant, respectively^16^). Therefore, the interaction of the finger domains within IMPDH octamers described in this work for AgIMPDH and by Labesse *et al*.^16^ for PaIMDH might constitute a conserved mechanism that regulates catalytic activity in response to ligand binding to the adjacent Bateman domain.

Despite the clear inhibitory role we have demonstrated for GTP and GDP in the eukaryotic IMPDHs we have analysed, the precise molecular mechanisms and the role of Mg-ATP activation^16^ and/or oligomerization^27^ in bacterial IMPDHs remain unknown. According to a very recent classification^27^, the bacterial enzymes we have analysed (EcIMPDH and BsIMPDH) would belong to a ‘class II' of tetrameric IMPDHs, whose catalytic activity is not significantly affected by Mg-ATP^27^. Nevertheless, the mechanism of activation by Mg-ATP in ‘class I' octameric IMPDHs (where PaIMPDH is included) is not yet defined and further experiments are required to clarify this issue. In any case, the differences observed in the allosteric regulation open the door to a promising unexplored strategy to differentially target eukaryotic and prokaryotic IMPDHs, different from the previously proposed approaches^22^,^24^,^30^.

At this point, we do not know whether AgIMPDH octamers merely represent an inhibited state of the enzyme or if they have other functions *in vivo.* It is tempting to speculate that IMPDH might act as a sensor of the intracellular levels of GTP, changing its oligomeric state accordingly and coupling the cell energy status (that is, GTP/GMP ratio) to the different IMPDH's moonlighting functions, such as repression of the transcription of proliferating genes, as has been recently described in *Drosophila*^18^. Experiments are currently ongoing at our laboratory to further investigate this intriguing possibility.

**Figure 6:**
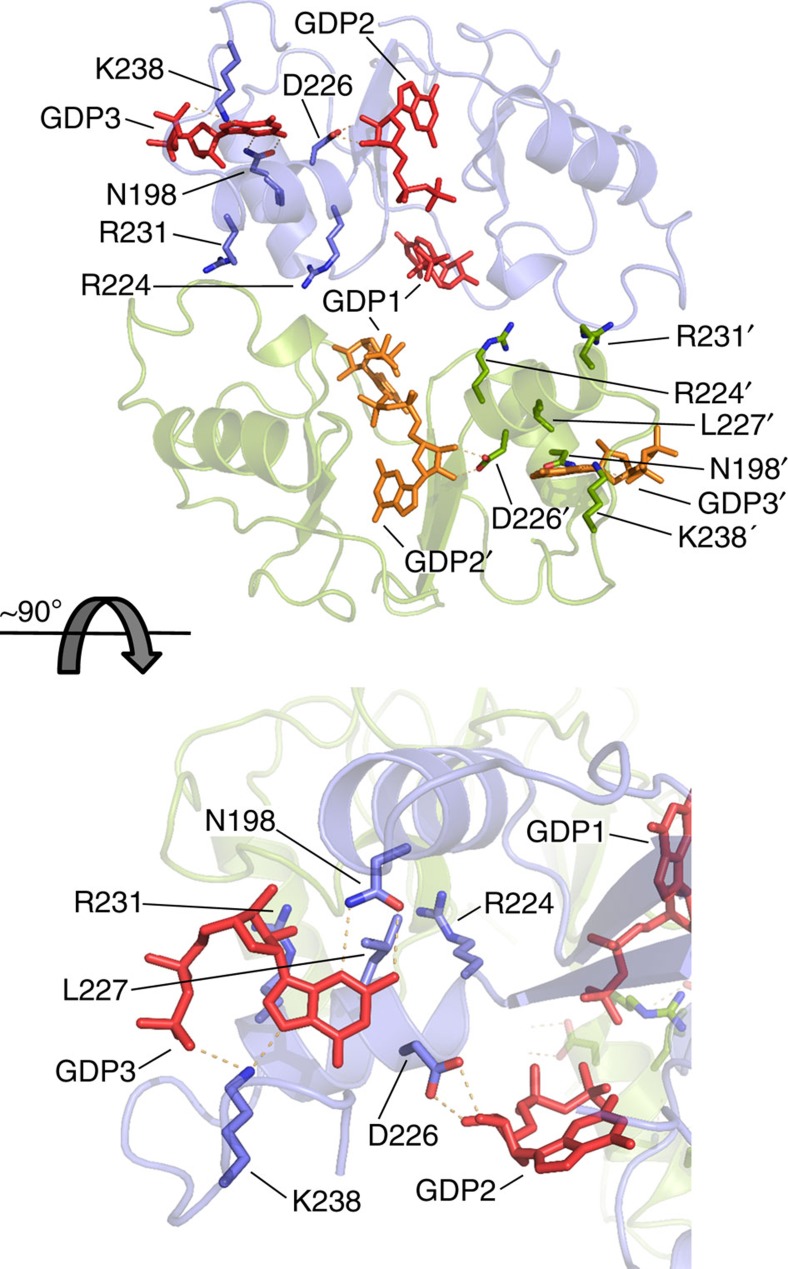
Missense mutations of HsIMPDH1 linked to retinopathies map into the nucleotide-binding sites. Blue and green cartoon representations of two interacting Bateman domains of a HsIMPDH1 homology model bound to GDP (red or orange sticks). The side chain of residues whose mutations are linked to RP and LCA are shown in sticks. Key interactions are represented by orange dashes.

Our finding that HsIMPDH1 is sensitive to GTP/GDP allosteric inhibition is especially interesting, because several mutations in HsIMPDH1 are associated with either autosomal dominant RP (adRP^13^,^25^,^31^,^32^,^33^; T116M, R224P, D226N, L227P, R231P, K238E/K238R, V268I and H372P) or the more severe retinal degeneration, LCA^13^ (R105W and N198K). Strikingly, six of these mutations correspond to residues in the CBS2 motif that lie in the guanine nucleotide-binding sites or are at the interface between the Bateman domains. In fact, after mapping these residues in a homology model of HsIMPDH1 bound to GDP (built based on our AgIMPDH-GDP structure), it was observed that Arg224 was directly involved in the binding of GDP2, whereas residues Asp226 and Arg231 participated in the interface of the Bateman domains (Fig. 6). Moreover, residues Asn198, Leu227 and Lys238 interacted directly with GDP3 at the non-canonical site (Fig. 6), further supporting our hypothesis that this site indeed has a relevant physiological role. Thus, these observations predict that the HsIMPDH1 mutations associated with adRP and LCA will result in enzymes that cannot be inhibited by GTP and/or GDP, that is, they are constitutively active. This prediction is reinforced by data showing that the equivalent mutations of Arg226 and Asn198 in AgIMPDH (this work) or Asp224 in PaIMPDH^16^ abrogate nucleotide binding to the Bateman domain and the subsequent allosteric signal. As HsIMPDH1 is mainly responsible for the bulk GTP supply to the photoreceptor cells^34^, the deregulation of HsIMPDH1 might indeed result in altered pools of purine nucleotides. This is especially important in the retina, one of the most energy-demanding tissues, with the highest metabolic rate of the human body, full of photoreceptors that require GTP for visual transduction processes^35^. Furthermore, our hypothesis would perfectly explain the dominant character of the IMPDH1 mutations associated with these pathologies, as the loss of GTP regulation in one of the two alleles would be sufficient to account for the altered nucleotide pools and cause a disorder. Taken together, our results suggest the potential involvement of the allosteric regulation of HsIMPDH1 by GTP and GDP in adRP and LCA pathologies, and hint at new research lines that deserve further exploration, as they could provide a new and promising mechanistic framework for drug discovery.

## Methods

### *A. gossypii* strains

All the strains used in this work were built using the previously constructed *ΔIMPDH* strain as parental^6^. The strains *ΔBateman*, *HA-IMPDH* and *HA-ΔBateman* were constructed by using DNA replacement cassettes containing the open reading frame of interest and flanking homology regions specific for integration within the *IMPDH* gene. In *ΔBateman*, the whole Bateman domain (residues Tyr116–Tyr235) has been replaced by the sequence SQDG. In the HA-tagged constructs, the HA epitope (GSG-YPYDVPDYA-GSG) was inserted within residues Ala242 and Asp243 of either wild-type AgIMPDH or ΔBateman proteins. The DNA replacement cassettes were transformed into spores of the *ΔIMPDH* strain and the transformants, prototroph for guanine in contrast to the parental strain (*ΔIMPDH*), were selected in minimal media. Homokaryotic transformants were obtained after sporulation and clonal selection of the primary heterokaryotic transformants.

The *P*_*GPD*_*-ΔBateman* strain was obtained as follows: the ΔBateman open reading frame was inserted into a DNA cassette comprising a recombination module for stable integration into the *STE12* locus and an overexpression module based on the *A. gossypii* glycerol 3-phosphate dehydrogenase promoter (*P*_GDP_) and terminator sequences, which have been reported to provide constitutive and high-expression transcription levels^36^,^37^. This DNA cassette was transformed into spores of the *ΔIMPDH* strain and the transformants selected as described above. All strains were checked for correct integrations by PCR amplification and DNA sequencing.

### Media and culture of *A. gossypii*

*A. gossypii* ATCC 10895 was used as the wild-type strain in this work. *A. gossypii* cultures were grown at 28 °C (150 r.p.m. orbital shaking) in rich MA2 medium^38^. When required, a concentration of 250 μg ml^−1^ of geneticin (G418) (Sigma-Aldrich) was used. *A. gossypii* transformation, sporulation, spore isolation and nucleic acid isolation were performed using standard, well-stablished methodologies^37^,^39^,^40^.

### Protein purification

Modified pET15b bacterial expression DNA plasmids^41^ for AgIMPDH and ΔBateman proteins were obtained as described^6^. Human and bacterial IMPDHs were obtained from a complementary DNA library and from genomic DNA, respectively, and cloned into the modified pET15b expression vector. Point mutations were introduced by site-directed mutagenesis using the methodology described in the QuikChange II method (Agilent Technologies). All DNA constructs were corroborated by DNA sequencing. Bacterial and fungal IMPDHs were expressed in *E. coli* strain BL21 (DE3) and purified by nickel-chelating affinity chromatography according to standard protocols. The His_8_ tag present at the amino terminus of the fusion proteins was cleaved by digestion with tobacco etch virus protease (His_6_ tagged) and the protease, together with residual uncleaved protein, was removed by a second nickel-affinity chromatography. The cleaved protein was then injected into a HiPrep Sephacryl S-300 16/60 HR size-exclusion chromatography column (GE Healthcare Life Sciences) equilibrated in buffer 20 mM Tris-HCl, 150 mM KCl, 0.5 mM TCEP (Tris(2-carboxyethyl)phosphine) pH 8.0. The two human isoforms were prepared as described before^20^. Fractions containing AgIMPDH proteins were pooled, concentrated in a 10-kDa cutoff Amicon Ultra Centrifugal Filter (Millipore), flash-frozen into liquid nitrogen and stored at −80 °C until use. All the enzymes analysed did not significantly lose activity after thawing.

### Metabolite analysis

Extracellular inosine concentration were determined from the *A. gossypii* culture broth by HPLC. Briefly, mycelia from 5 ml culture broth were harvested by filtration on filter paper, dried overnight at 100 °C and weighed. Filtered medium was passed through a 0.2μm polyvinylidene difluoride membrane (Acrodisc LC; Pall Life Sciences) and injected into an AQUASIL C18 140 × 4.6 mm column (Thermo Fisher Scientific) connected to an HPLC device (Agilent 1120 Compact LC), to determine extracellular nucleoside concentrations by monitoring absorbance at 260 nm. The separation of nucleosides was achieved by using an isocratic flow of phosphate buffer, pH 5.5, plus 0.5% of acetonitrile. Quantification was carried out using a calibration curve prepared with pure standards of inosine (Sigma-Aldrich). All analyses were performed using three biological replicates.

### Chemical cross-linking

Samples of AgIMPDH or ΔBateman at 2 mg ml^−1^ in the presence of the different nucleotides were cross-linked with 1 mM disuccinimidyl suberate (spacer length, 11.4 Å; Pierce, Thermo Fisher Scientific) in buffer A: 20 mM HEPES, 100 mM KCl, 1 mM dithiothreitol (DTT) pH 8.0, at room temperature for 30 min. The reaction was quenched by adding 50 mM Tris-HCl for 15 min at room temperature. The resulting samples (7.5 μl) were analysed in a 4–15% gradient SDS–PAGE (BioRad). Cellular protein extracts were obtained by resuspending the mycelia of the different strains (24 h after inoculating, 500 μl spores in 100 ml MA2 medium and incubating at 28 °C with orbital shaking, that is, cultures in the exponential growth phase) in buffer A supplemented with a protease inhibitor cocktail (cOmplete Protease Inhibitor Tablets, Roche) and disrupted with a French Press. After centrifugation, whole extracts were cross-linked as described above and subjected to western blot analysis using a rat monoclonal anti-HA antibody coupled to horseradish peroxidase (Roche), following the manufacturer's instructions.

### IMPDH activity assay and enzyme kinetics

IMPDH activity was assayed by monitoring the reduction of NAD^+^ to NADH and the subsequent increase in absorbance at 340 nm in buffer: 100 mM Tris-HCl, 100 mM KCl, 2 mM DTT pH 8.0. Final enzyme concentrations were set between 20 and 100 μg ml^−1^, the NAD^+^ concentration was fixed at 0.5 mM, IMP varied from 0.019 to 5 mM and inhibitors were assayed at concentrations ranging from 0.02 to 5 mM. When Mg^+2^ was included in the reaction, the total concentration of MgCl_2_ was 5 mM. The experimental data were fitted to the Michaelis–Menten equation of enzyme kinetics using the OriginPro software (OriginLab Corporation), to obtain the maximum apparent initial velocity (*V*_max_^app^) and the apparent Michaelis–Menten constant (*K*_M_^app^):


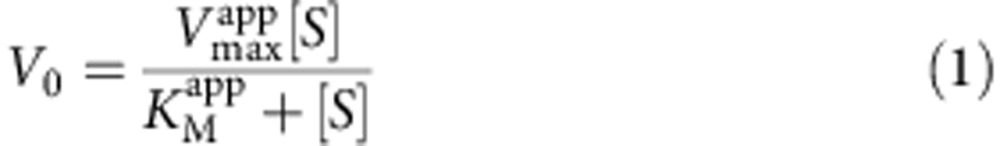


Following this equation, the global nonlinear regression analysis of *V*_max_^app^ and *K*_M_^app^, as a function of the inhibitor concentration, allowed the intrinsic Michaelis–Menten constant (*K*_M_) and inhibition constant (*K*_i_) for competitive inhibition to be determined. The substrate–inhibitor heterotropic cooperative interaction and the fractional activity of the ternary enzyme–substrate–inhibitor complex, *α* and *β* respectively, were estimated using a mixed inhibition model:


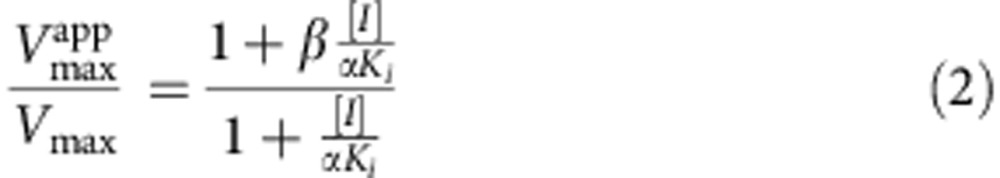



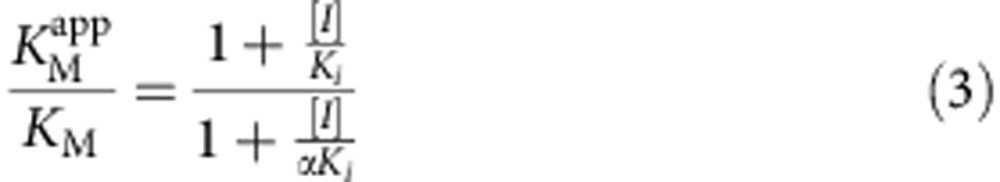


The constant *α* reflects the effect of the inhibitor on the apparent affinity of the enzyme for the substrate. Several scenarios are possible: (1) an *α*=1 indicates that there is no effect (substrate and inhibitor bind independently); (2) an *α*=infinity indicates that the binding of the inhibitor is competitive with respect to the substrate (that is, the ternary complex is not allowed); and (3) an *α* > 1 indicates that inhibitor binding decreases the apparent affinity of the enzyme for its substrate.

On the other hand, the *β*-factor reflects the modification of the rate of product formation caused by the inhibitor: (1) a *β*=0 indicates that the ternary complex has no activity; (2) a *β* < 1 indicates that the ternary complex has lower activity than the enzyme–substrate complex (that is, the inhibitor partially blocks product formation); and (3) a *β* > 1 indicates that the ternary complex has higher activity than the enzyme–substrate complex (that is, the enzyme is activated).

### Isothermal titration calorimetry

Standard ITC experiments were performed using an AutoITC200 system (Malvern Instruments). Briefly, a 20μM IMPDH solution in buffer 20 mM HEPES, 100 mM KCl, 2 mM DTT pH 8.0, was titrated at 25 °C with a 300μM IMP solution. The resulting heats were integrated and fitted to a single binding site model implemented in the software package Origin (OriginLab Corporation).

### Small angle X-ray scattering

SAXS measurements were performed at the P12 beamline at EMBL-Hamburg. Buffer (20 mM Tris-HCl pH 8.0, 150 mM KCl, 3 mM EDTA) and proteins at a concentration of 1.25, 2.5, 5, 10 and 20 mg ml^−1^ were measured in the presence or absence of 5 mM nucleotide. During the measurements, both buffer and proteins were flowing through a 1.6-mm diameter quartz capillary to minimize radiation damage and 20 frames of 50 ms each were recorded. The wavelength was 1.24 Å and the distance from the sample to the detector (Pilatus 2 M, Dectris) was 4.10 m, which allowed collecting data up to ∼0.35 Å^−1^ (4*π*sin*θ λ*^−1^) of the scattering vector. All data frames were averaged after checking each measurement for radiation damage. Buffer scattering was subtracted from protein samples and the resulting spectra were normalized by protein concentration and extrapolated to infinite dilution. The radius of gyration was calculated by the Guinier approximation^42^ and from the pair-distribution function, as implemented in GNOM^43^. The maximum interatomic distance (*D*_max_) was estimated from the pair-distribution function. The theoretical scattering profiles were calculated from the crystal structures using the programme CRYSOL^44^.

### Crystallization and structure determination

Crystals of the deletion mutant ΔBateman bound to GMP were grown at room temperature using vapour diffusion method by mixing a protein solution at 20 mg ml^−1^ in 10 mM Tris-HCl, 100 mM KCl, 10 mM GMP, 0.25 mM TCEP pH 8.0, with an equal volume of mother liquor consisting of 25% (v/v) 1,2-propanediol, 5% (w/v) PEG-3000, 10% (v/v) glycerol, 0.1 M sodium phosphate citrate pH 4.2. Crystals of AgIMPDH complexed to GDP were obtained as before in mother liquor: 40% (v/v) 1,2-propanediol, 0.1 M sodium acetate pH 4.5, after adding up to 10 mM GDP to a 22 mg ml^−1^ AgIMPDH protein solution.

Protein crystals were flash-cooled in liquid nitrogen and data were collected at 100 K, using monochromatic X-rays of 1.000 Å wavelength, at the PX-III beamline at SLS (Switzerland) and at the XALOC beamline^45^ in ALBA synchrotron (Spain). Diffraction intensities were indexed and integrated by using the software XDS and scaled with XSCALE^46^. Data were phased by molecular replacement using the programme PHASER^47^ within the CCP4 suite^48^, using as template the structure of *P. aeruginosa* IMPDH (PDB code 4AVF^49^). The structures were refined using the PHENIX crystallographic software package^50^, alternating manual modelling with COOT^51^. Simulated annealing (Cartesian coordinates), gradient-driven positional, restrained individual isotropic B-factor and TLS refinement^52^ were used for refinement of AgIMPDH-GDP. ΔBateman-GMP was refined using simulated annealing (Cartesian coordinates), gradient-driven positional and restrained individual anisotropic B-factors. Both structures have an excellent geometry with no Ramachandran outliers and 98% of the residues in favoured regions (2% in allowed regions). Stereo images of different portions of the electron density map for AgIMPDH-GDP and ΔBateman-GMP can be found in Supplementary Fig. 18.

A three-dimensional model for HsIMPDH1 was obtained by homology modelling with the Swiss-Model automated modelling server^53^, using the structure of AgIMPDH bound to GDP as template. The figures showing three-dimensional protein structures have been generated using PyMOL (The PyMOL Molecular Graphics System, Schrödinger, LLC).

### Prediction of SDPs

Protein sequences corresponding to the Bateman domain of IMPDH from *P. aeruginosa*, *A. gossypii* and *L. donovani* were used independently to retrieve homologous sequences from the rp75-UniProt representative set in HMMER using the phmmer search tool^54^. Each set was further filtered to reject sequences >90% identical. The remaining sequences, containing 110 representative non-redundant IMPDH Bateman sequences, were aligned with MUSCLE^55^. The resulting multiple sequence alignment was analysed interactively to discover potential SDPs using the S3Det method^56^ implemented in the JDet software package^57^.

## Additional information

**Accession codes:** The atomic coordinates and the structure factors of AgIMPDH-ΔBateman-GMP and AgIMPDH-GDP have been deposited in the Research Collaboratory for Structural Bioinformatics Protein Data Bank under ID codes 4Z0G and 4Z87, respectively.

**How to cite this article:** Buey, R. M. *et al*. Guanine nucleotide binding to the Bateman domain mediates the allosteric inhibition of eukaryotic IMP dehydrogenases. *Nat. Commun.* 6:8923 doi: 10.1038/ncomms9923 (2015).

## Supplementary Material

Supplementary Information Supplementary Figures 1-19, Supplementary Table 1, Supplementary Note 1 and Supplementary References

## Figures and Tables

**Table 1:** Enzyme kinetic inhibition constants.

	***K***_**M**_ **(μM)**		***K***_**i**_**(mM)**	***α***	***β***	**Inhibition**
AgIMPDH	51±8	GMP	0.60±0.1	−	−	Competitive
		GDP	0.21±0.04	6.5±0.8	0.033±0.005	Mixed type
		GTP	0.16±0.04	4.1±0.7	0.24±0.05	Mixed type
HsIMPDH1	41±2	GMP	0.96±0.08	−	−	Competitive
		GDP	0.23±0.04	4.7±0.7	0.12±0.04	Mixed type
		GTP	0.54±0.06	3.8±0.5	0.11±0.04	Mixed type
HsIMPDH2	31±4	GMP	1.3±0.2	−	−	Competitive
		GDP	0.61±0.06	6.6±0.8	0.14±0.05	Mixed type
		GTP	0.48±0.06	3.0±0.4	0.51±0.08	Mixed type
BsIMPDH	120±20	GMP	0.42±0.07	−	−	Competitive
		GDP	2.3±0.4	2.9±0.4	0.8±0.1	Mixed type—weak
		GTP	−	−	−	No
EcIMPDH	80±10	GMP	1.1±0.1	−	−	Competitive
		GDP	−	−	−	No
		GTP	0.68±0.07	−	−	Competitive

GDP, guanosine-5′-diphosphate; GMP, guanosine-5′-monophosphate; GTP, guanosine-5′-triphosphate.

Values are given with s.e. The type of inhibition is shown in the column on the far right. The parameter α represents the substrate–inhibitor heterotropic cooperative interaction and β is the fractional activity of the ternary enzyme–substrate-inhibitor complex, as described in the Methods section.

**Table 2:** SAXS-derived hydrodynamic parameters.

	**Rg (Å)**	***D***_**max**_ **(Å)**	**Porod's volume (Å**^**3**^**)**	**Estimated MW (kDa)**	**Theoretical MW (kDa)**
WT-APO	45.1±0.2	158	401.504	241	228 (Tetramer)
WT-GMP	44.0±0.3	154	367.818	220	
WT-GDP	49.7±0.2	159	807.586	485	456 (Octamer)
WT-GTP	49.6±0.3	163	790.699	475	
ΔBateman-APO	38.2±0.2	128	288.670	173	176 (Tetramer)
ΔBateman-GMP	36.8±0.4	121	298.568	179	

MW, molecular weight; SAXS, small angle X-ray scattering.

Hydrodynamic parameters estimated from the SAXS data in the presence or absence of 5 mM guanine nucleotides. Rg values are given with s.e.

**Table 3:** X-ray crystallography data collection and refinement statistics.

	**AgIMPDH-GDP**	**AgIMPDH-ΔBateman-GMP**
*Data collection*		
** **Space group	P4	P4
*Cell dimensions*		
*a,b,c* (Å)	122.0, 122.0, 147.6	117.5, 117.5, 56.5
*α*, *β*, *γ* (°)	90.0, 90.0, 90.0	90.0, 90.0, 90.0
Resolution (Å)	45.63–2.25 (2.33–2.25)	52.54–1.25 (1.295–1.25)
** ***R*-merge	0.104 (1.527)	0.055 (1.515)
*I*/*Iσ*	25.70 (1.97)	22.78 (1.34)
Completeness (%)	100 (100)	100 (99)
Unique reflections	102,241 (10188)	212,419 (20,923)
Redundancy	13.8 (14.2)	12.9 (10.4)
*Refinement*		
Resolution (Å)	2.25	1.25
No. reflections	102,236 (10,187)	212,413 (20922)
*R*-work	0.203 (0.301)	0.128 (0.325)
*R*-free	0.223 (0.320)	0.149 (0.333)
No. atoms	15,525	6,545
Macromolecules	14,666	5,886
Ligand/ion	859	659
Water	447	645
*B-factors*		
Macromolecules	55.3	19.8
Ligands	57.2	48.7
Water	44.3	32.9
*Root mean squared deviations*		
Bond lengths (Å)	0.014	0.031
Bond angles (°)	1.18	1.68
		
*Ramachandran statistics*		
Favoured (%)	98	98
Allowed (%)	2	2
Outliers (%)	0	0
Rotamer outliers (%)	1	1

Statistics for the highest-resolution shell are shown in parentheses. Friedel mates were averaged when calculating reflection statistics. Data for both structures were collected using a single crystal.

**Table 4:** ITC-derived thermodynamic parameters.

	***K***_**d**_**(μM)**	**Δ*****G*** **(kcal** **mol**^**−1**^**)**	**Δ*****H*** **(kcal** **mol**^**−1**^**)**	**−*****T*****Δ*****S*** **(kcal** **mol**^**−1**^**)**
IMPDH	1.7	−7.9	−26.4	18.5
IMPDH + GTP	26	−6.3	−55.0	48.7
IMPDH + GDP	63	−5.7	−30.9	25.2

GDP, guanosine-5′-diphosphate; GTP, guanosine-5′-triphosphate; IMP, Inosine-5′-monophosphate; IMPDH, IMP dehydrogenase; ITC, isothermal titration calorimetry.

IMP titration of different AgIMPDH wild-type protein in the presence or absence of 5 mM GTP or GDP. Estimated relative error in *K*_d_ is 15–20%, absolute error for Δ*G* is ∼0.1 kcal mol^−1^ and the absolute error for Δ*H* and –*T*Δ*S* is 0.3–0.5 kcal mol^−1^.
