# Supplementary material for: Guanine nucleotide binding to the Bateman domain mediates the allosteric inhibition of eukaryotic IMP dehydrogenases
Source: Nat Commun. 2015 Nov 12;6:8923. doi: 10.1038/ncomms9923 (PMC4660370; doi:10.1038/ncomms9923)
Supplement: Supplementary Information — Supplementary Figures 1-19, Supplementary Table 1, Supplementary Note 1 and Supplementary References [file ncomms9923-s1.pdf]

SUPPLEMENTARY FIGURES

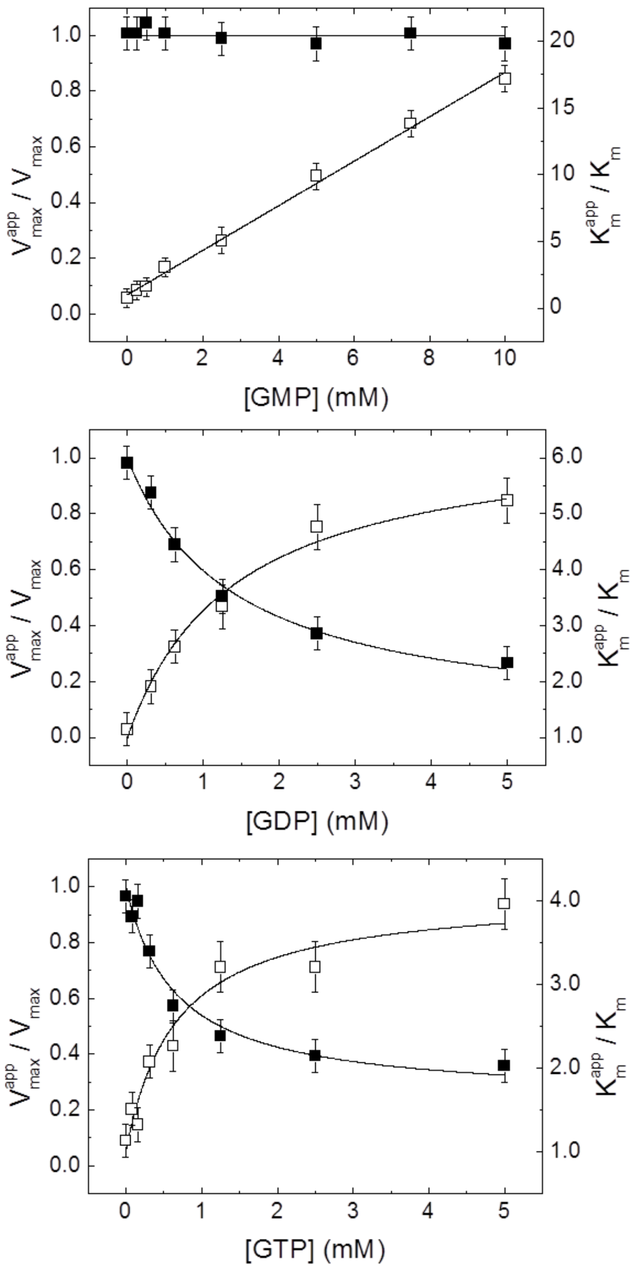

Supplementary Figure 1. *Inhibition of the catalytic activity of AgIMPDH by guanine nucleotides*. The plots represent the values of V_{max}^{app} / V_{max} (black squares) and K_M^{app} / K_M (white squares) at different concentrations of GMP (upper panel), GDP (middle panel) and GTP (lower panel). Reaction buffer (100 mM TrisHCl, 100 mM KCl, 2 mM DTT, pH 8.0) did not contained Mg^{+2}. The V_{max}^{app}, V_{max}, K_M^{app} and K_M values were obtained by fitting the initial velocities calculated from the time-course enzyme kinetic data to the Michaelis-Menten equation. The error bars represent the standard errors.

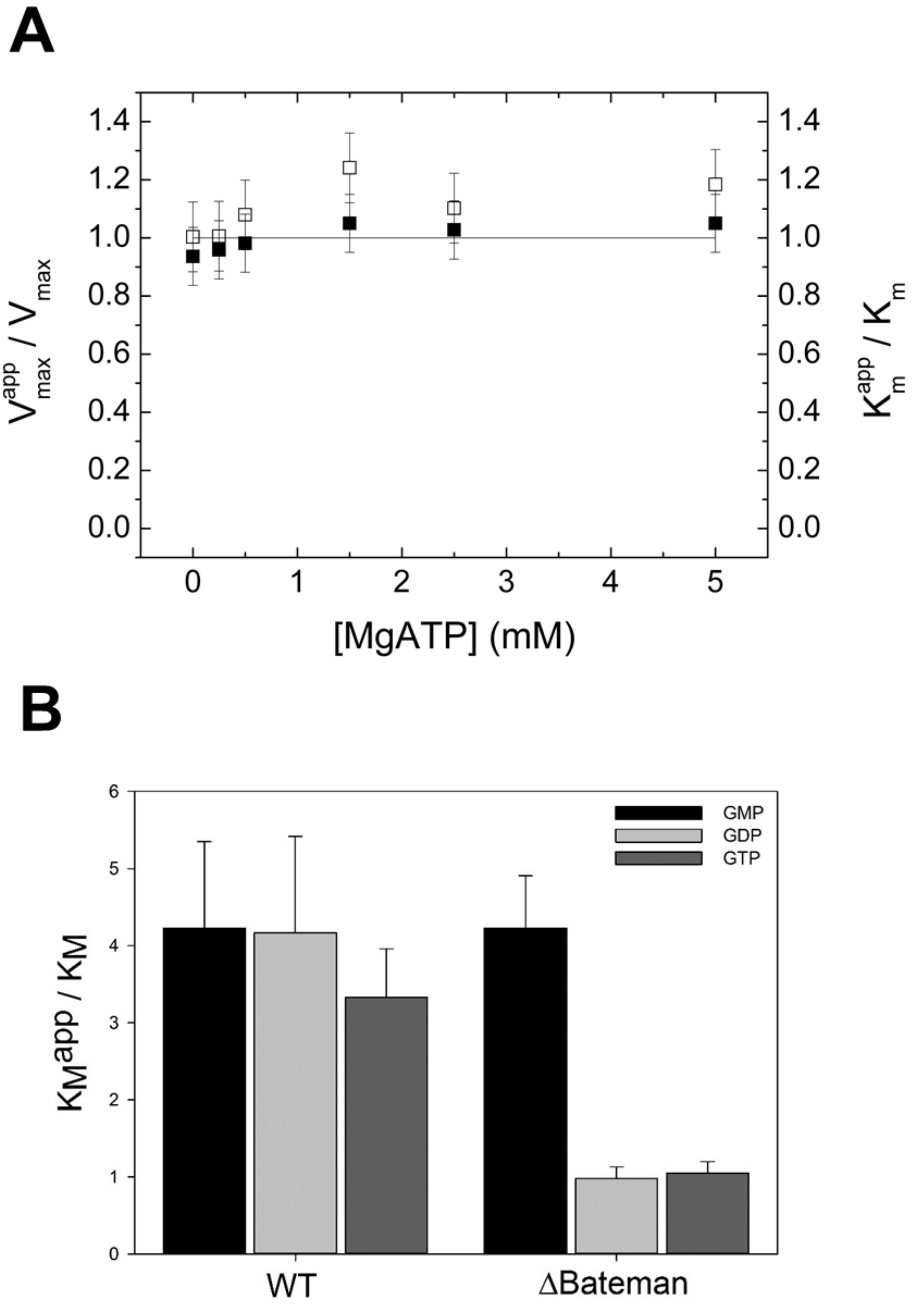

Supplementary Figure 2. *Catalytic activity of AgIMPDH is not affected by MgATP*. **A.** The plot shows the insignificant variation of the values of V_{max}^{app} / V_{max} (black squares) and K_M^{app} / K_M (white squares) with the indicated concentrations of ATP. Total $MgCl_2$ concentration was 5 mM. **B.** Values of K_M^{app} / K_M in the presence of 2 mM GMP, GDP or GTP. The error bars represent the standard errors.

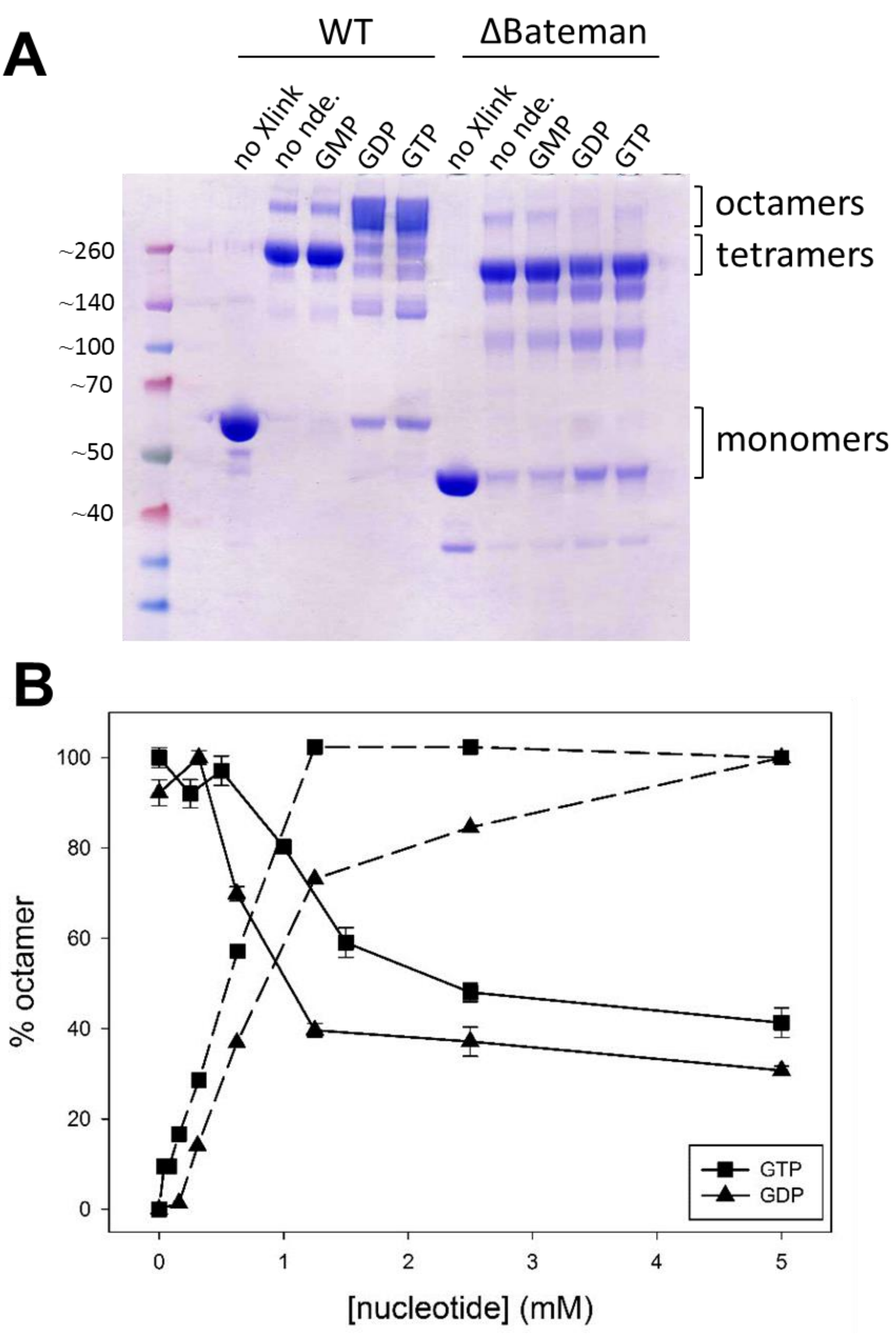

Supplementary Figure 3. *GDP and GTP alter the oligomeric state of AgIMPDH.* **A.** Coomassie Blue stained SDS-PAGE of cross-linked AgIMPDH and ΔBateman in the presence of 5 mM GMP, GDP or GTP. **B.** Plot representing the percentage of octamers in solution (dashed line) and the percentage of V^{app}_{max} (continuous line) as a function of the concentration GTP and GDP. The graph shows an inverse correlation between octamer formation and AgIMPDH catalytic activity.

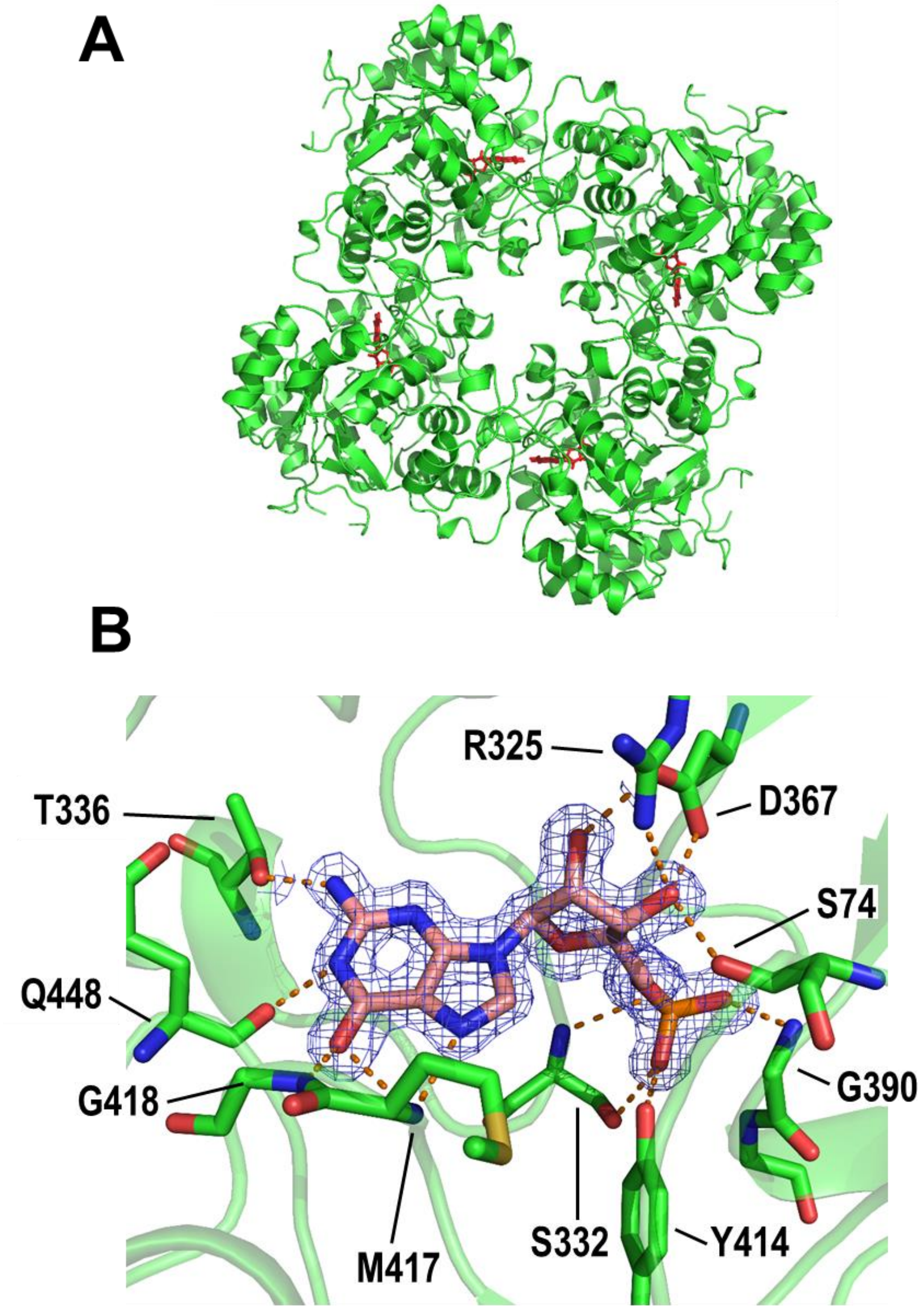

Supplementary Figure 4. *High resolution structure of ΔBateman bound to GMP*. **A.** Cartoon representation of the tetramer of ΔBateman (green cartoons) with GMP (red sticks) bound to the active site. **B**. Detailed view of GMP bound to the active site of AgIMPDH. Protein is shown in green ribbons; GMP and key interacting protein residues are shown in sticks. The blue mesh around GMP represents the simulated annealing omit $2mF_o$-DF_c electron density map contoured at 1σ level.

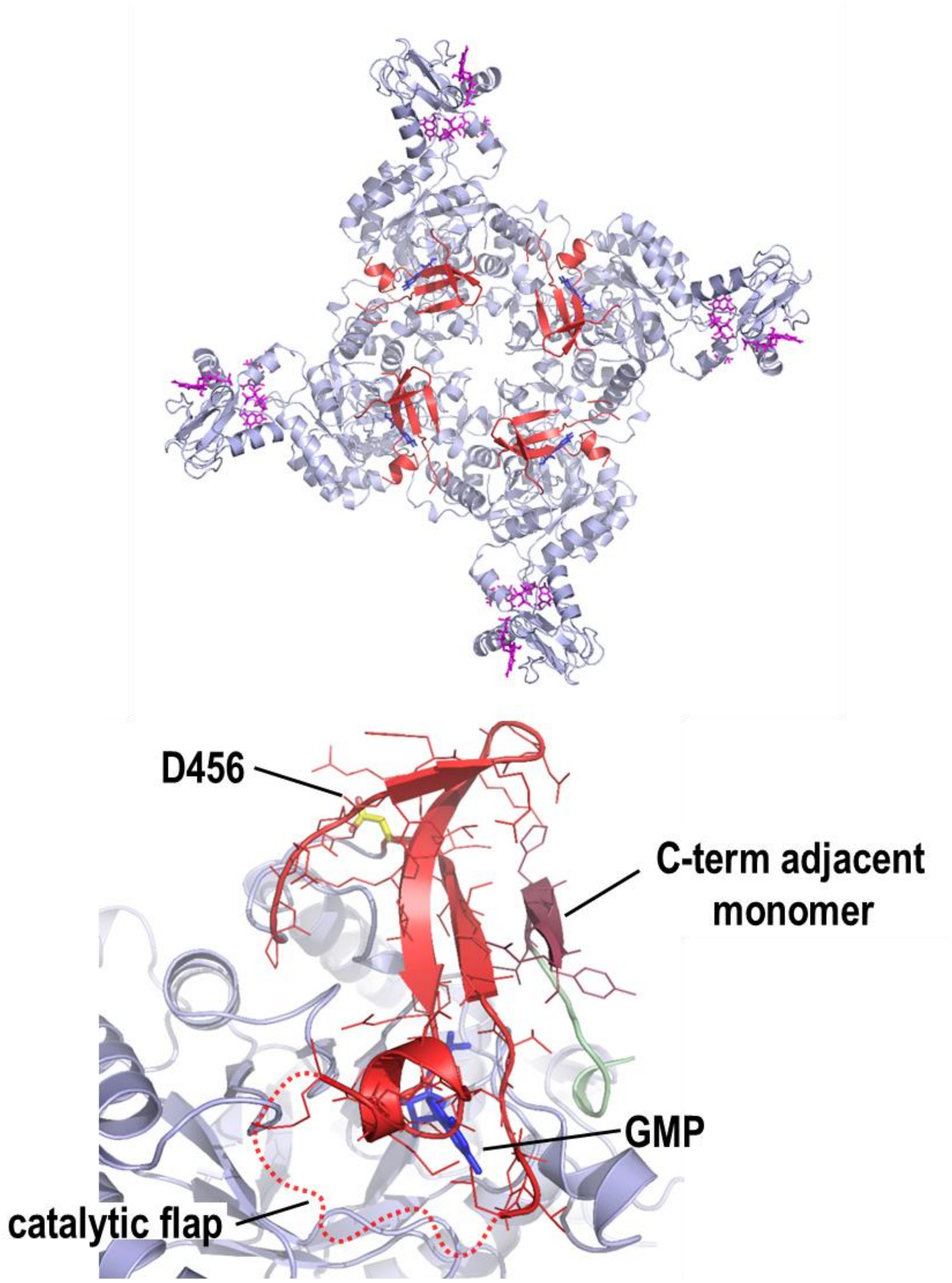

Supplementary Figure 5. *Structure of the finger domain of AgIMPDH*. Upper panel: cartoon representation of an AgIMPDH tetramer (light blue), with the finger domains coloured in red. GMP molecules bound to the active site are represented as blue sticks and GDP bound to the Bateman regulatory domain are shown in magenta sticks. Lower panel: close-up view of the structure of the finger domain represented as red cartoon and sticks. The side chain of residue Asp456 is shown in yellow sticks. The β-strand from the C-term of the adjacent monomer that completes the β-sheet that form the finger domain is shown in dark red. The catalytic flap (invisible in our structure) is represented as a discontinuous red line.

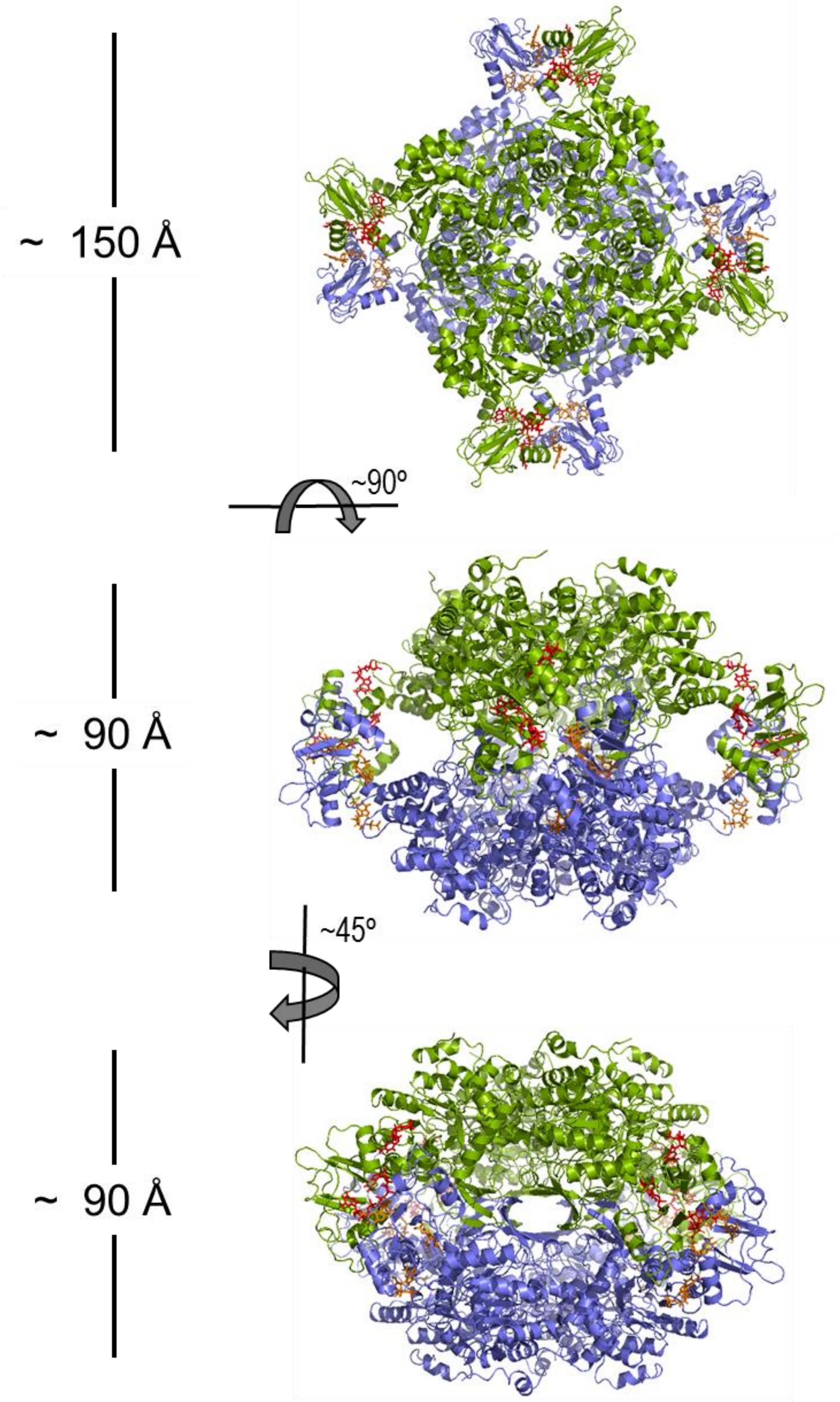

Supplementary Figure 6. *Structure of the octamers of AgIMPDH bound to GDP*. Different views of a cartoon representation of AgIMPDH octamers, obtained in the presence of GDP (red and orange sticks). The two tetramers that pile up tail to tail are colored green and blue. Approximate dimensions of the octamer are indicated on the left.

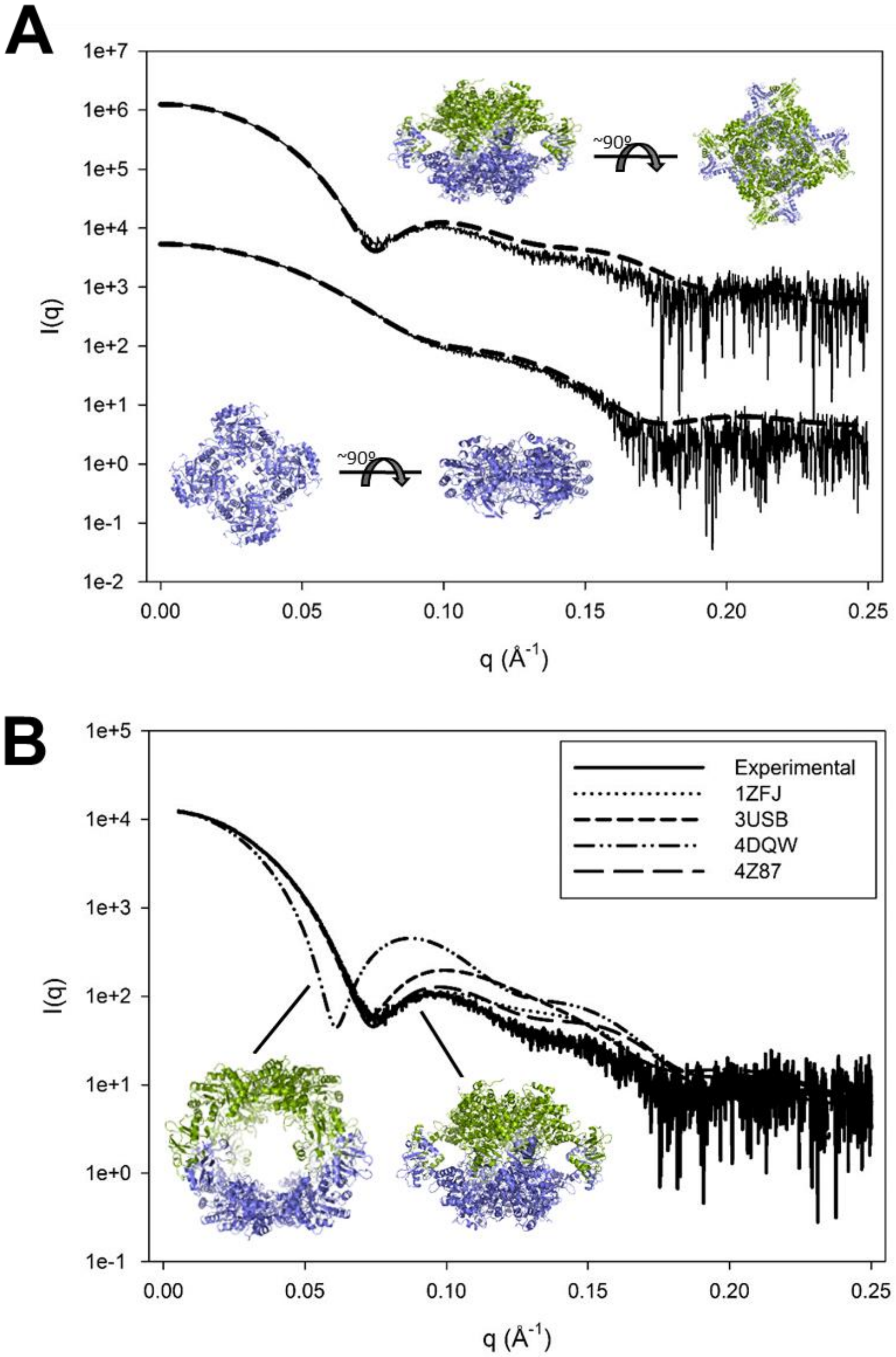

Supplementary Figure 7. *The crystal structures of AgIMPDH are identical to those in solution*. **A**. Experimental SAXS profiles (continuous lines) of the complexes AgIMPDH-GMP and AgIMPDH-GDP fitted to the theoretical ones (dashed lines) calculated from the high-resolution crystallographic structures. The SAXS profiles are vertically displaced to facilitate visualization. Cartoon representations of tetramers and octamers are shown within the graph. **B**. Experimental SAXS profiles of AgIMPDH-GDP octamers fitted to those ones calculated from the high-resolution crystallographic structures of *P. aeruginosa* (4DQW), *S. pyogenes* (1ZFJ), *B. anthracis* (3USB) and *A. gossypii* (4Z87).

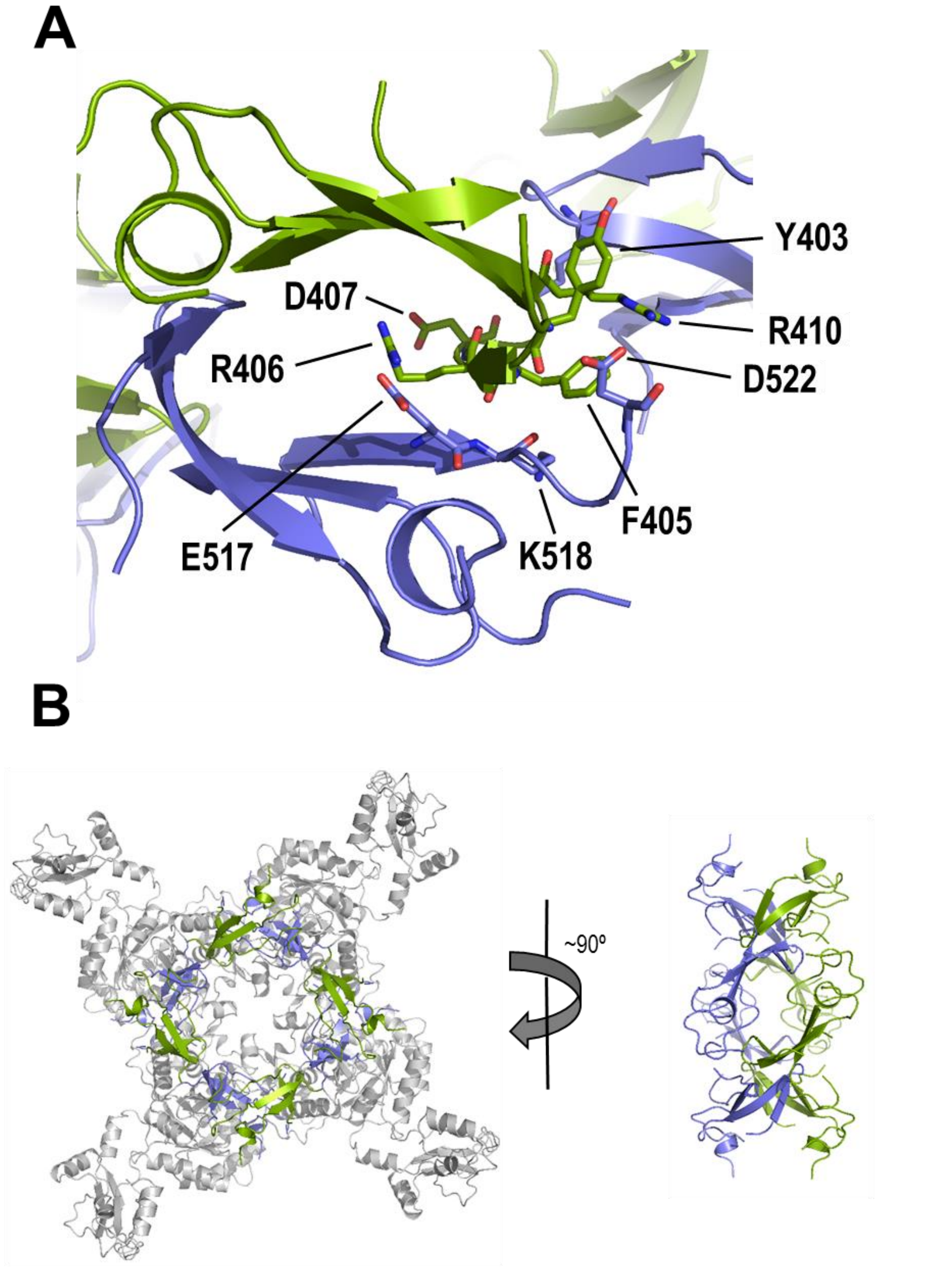

Supplementary Figure 8. *The finger domains of AgIMPDH interact within the octamers.* **A**. Cartoon representation of the interactions of the finger domain from two tetramers (green and blue) within the structure of AgIMPDH-GDP octamers. The side chains of key interacting residues are shown in sticks. **B**. Structure of IMPDH from S. pyogenes (PDB code 1ZFJ). The finger domains from the upper and lower tetramers are shown in green and blue cartoons around the quaternary (left) and binary symmetry axes (right).

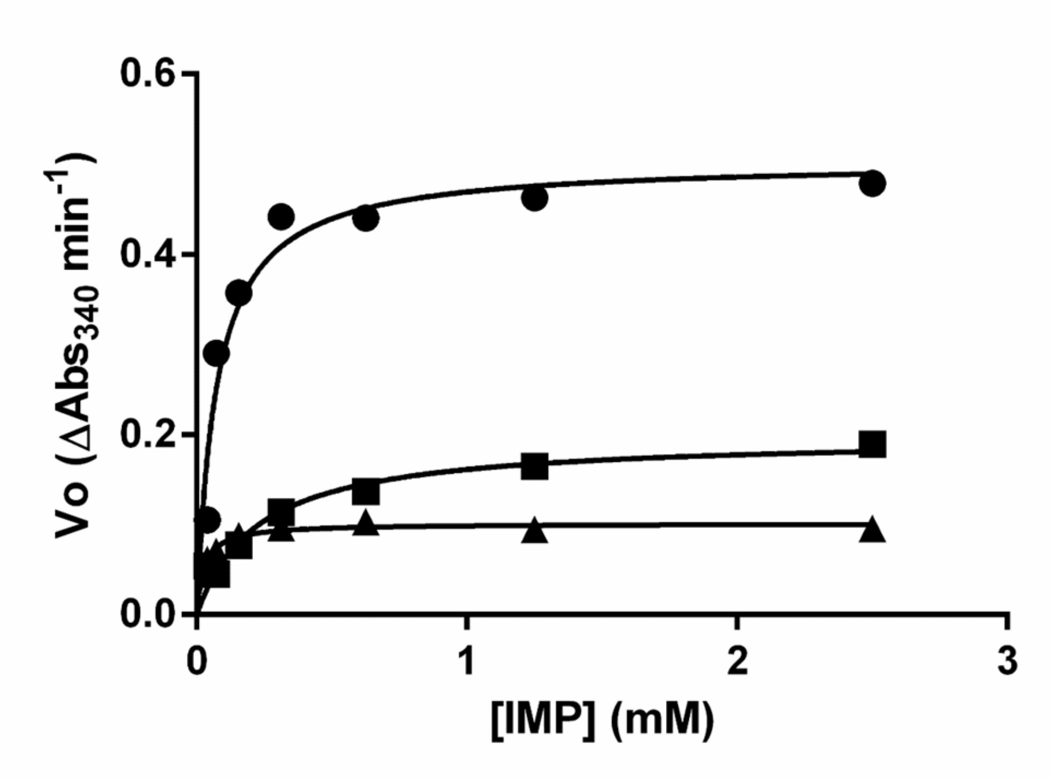

Supplementary Figure 9. *Mutations affecting the structure of the finger domain reduce catalytic activity.* Michaelis-Menten plot of the *in vitro* enzyme kinetics of wild-type AgIMPDH (circles) and mutants D456R (squares) and Δ513-522 (triangles). The continuous lines represent the non-linear regression fittings to Michaelis-Menten enzyme kinetics equation.

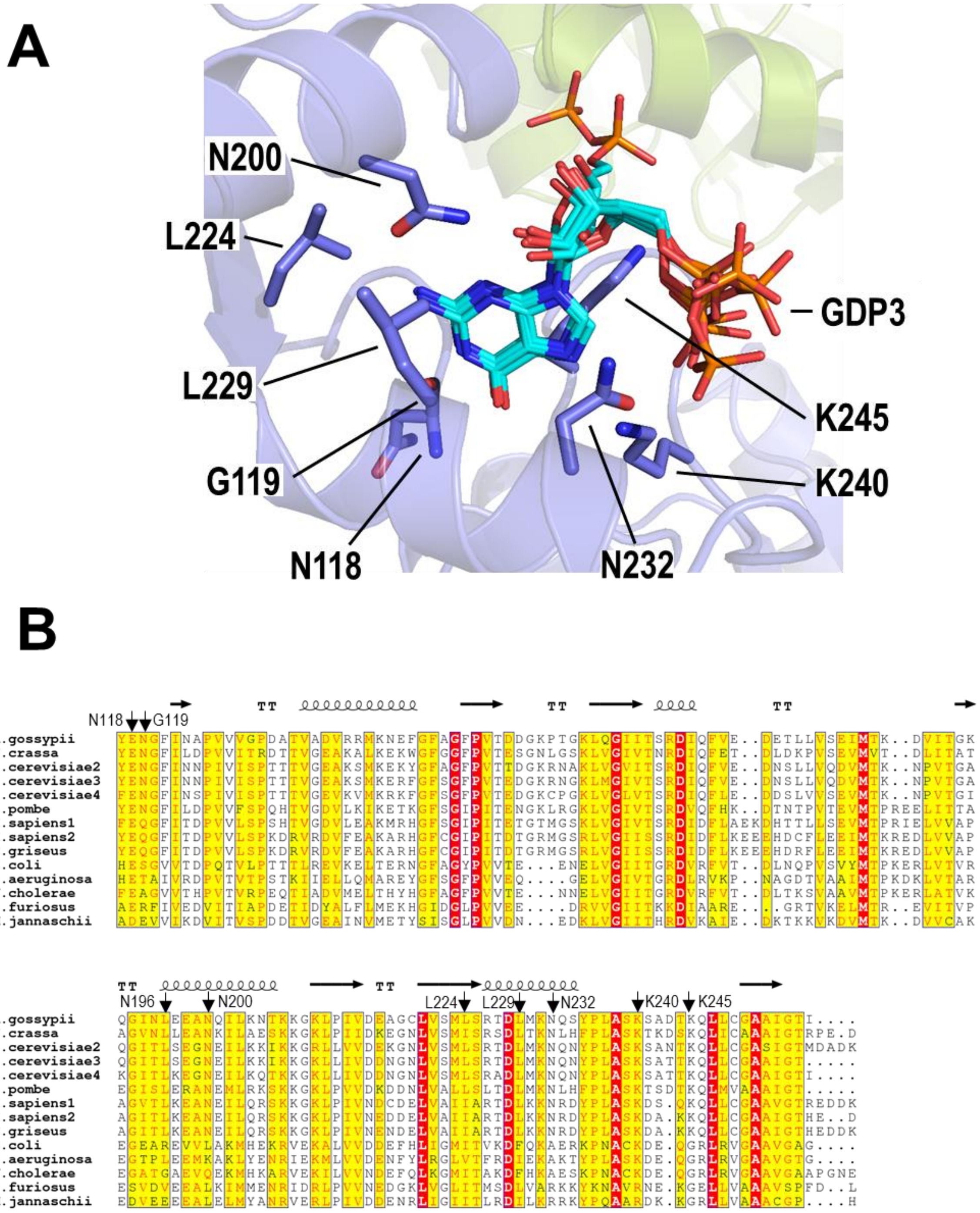

Supplementary Figure 10. *Sequence conservation and structure of the non-canonical guanine nucleotide binding site in the Bateman domain of AgIMPDH.* **A**. Superposition GDP3 molecules (in sticks) from chains B, C and D in the asymmetric unit of the AgIMPDH-GDP crystal into the GDP3 binding site of chain A. **B.** Multiple sequence alignment of the Bateman domain of different eukaryotic and prokaryotic IMPDHs. The key residues that interact with GDP in the non-canonical guanine nucleotide binding site are indicated.

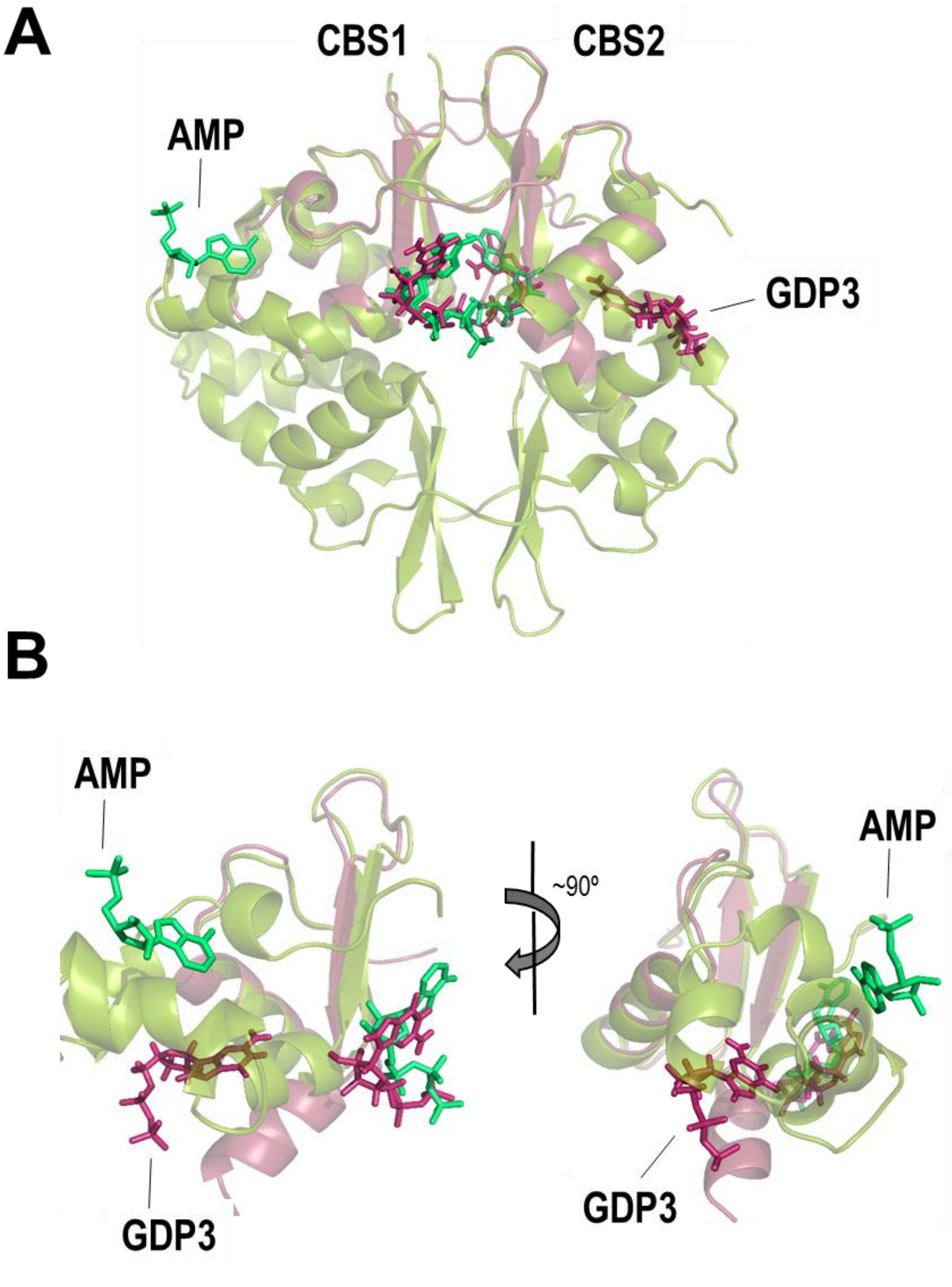

Supplementary Figure 11. Structural superimposition of the CBS module of AgIMPDH (red ribbons; PDB code 4Z87) and MJ1225 (green ribbons, PDB code 3LFZ). **A**. View of the whole CBS module of the chain A of both structures. **B**. Superimposition of the CBS1 motif from MJ1225 onto the CBS2 motif of AgIMPDH.

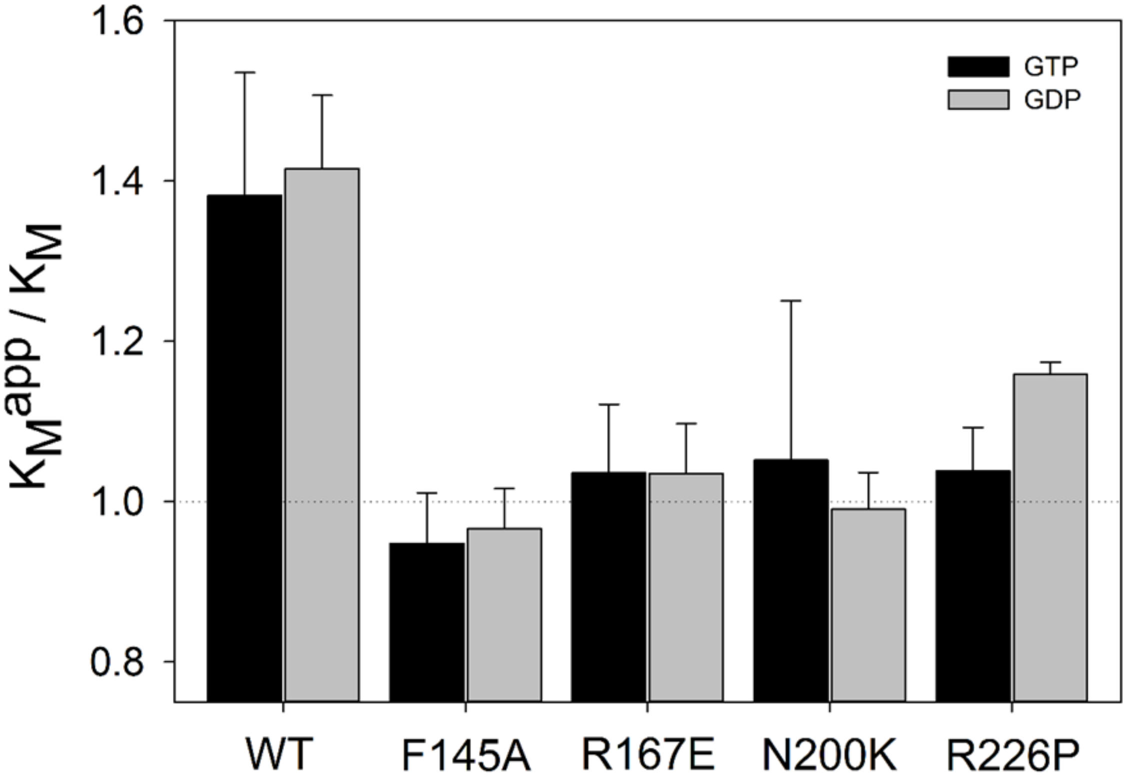

Supplementary Figure 12. *Mutations in the Bateman domain of AgIMPDH abrogate GTP/GDP-mediated allosteric inhibition.* Values of K_M^{app} / K_M in the presence of 1 mM GDP or GTP for different AgIMPDH mutants. The error bars represent the standard errors.

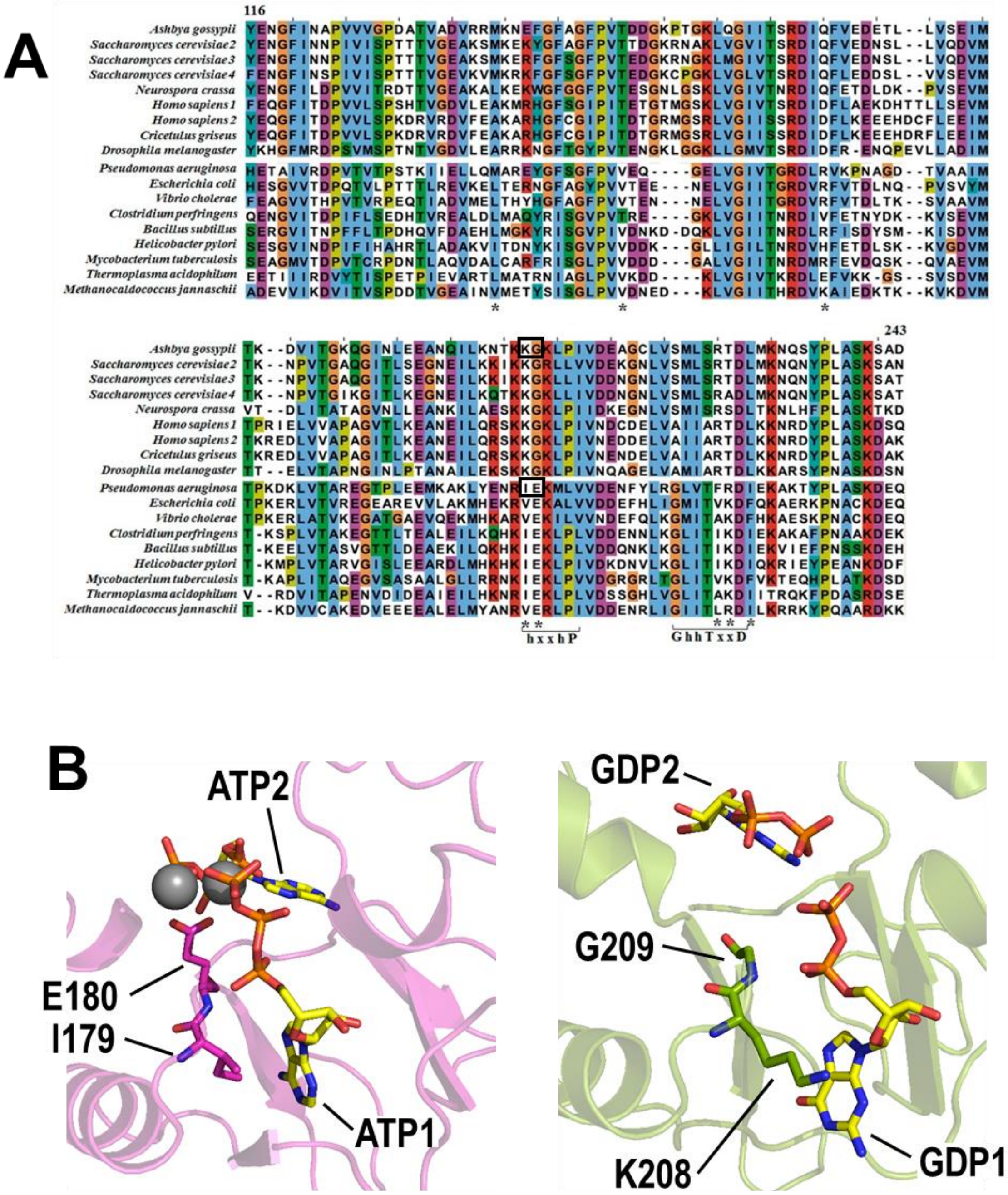

Supplementary Figure 13. *Specificity Determinant Positions (SDPs) in the Bateman domain of IMPDHs*. **A**. Multiple sequence alignment of the Bateman domain from different eukaryotic and prokaryotic IMPDHs. SDPs are marked with asterisks. The canonical nucleotide binding motifs ("h-x-x-h-P" and "G-h-h-T-x-x-D"[1, 2]) in CBS2 are indicated. **B.** Detailed view of the "RIEK" (left) and "KKGK" (right) sequence motifs that define the binding of MgATP or GTP/GDP to the Bateman domains of IMPDHs. The left panel shows PaIMPDH (PDB code 4DQW) in pink cartoons, ATP in sticks and manganese atoms as solid grey spheres. The right panel shows a similar view for AgIMPDH (PDB code 4Z87), in green cartoons, and GDP, in sticks. Residues Ile179/Glu180 from the "RIEK" motif (left) and Lys208/Gly209 from the "KKGK" motif (right) are shown in sticks.

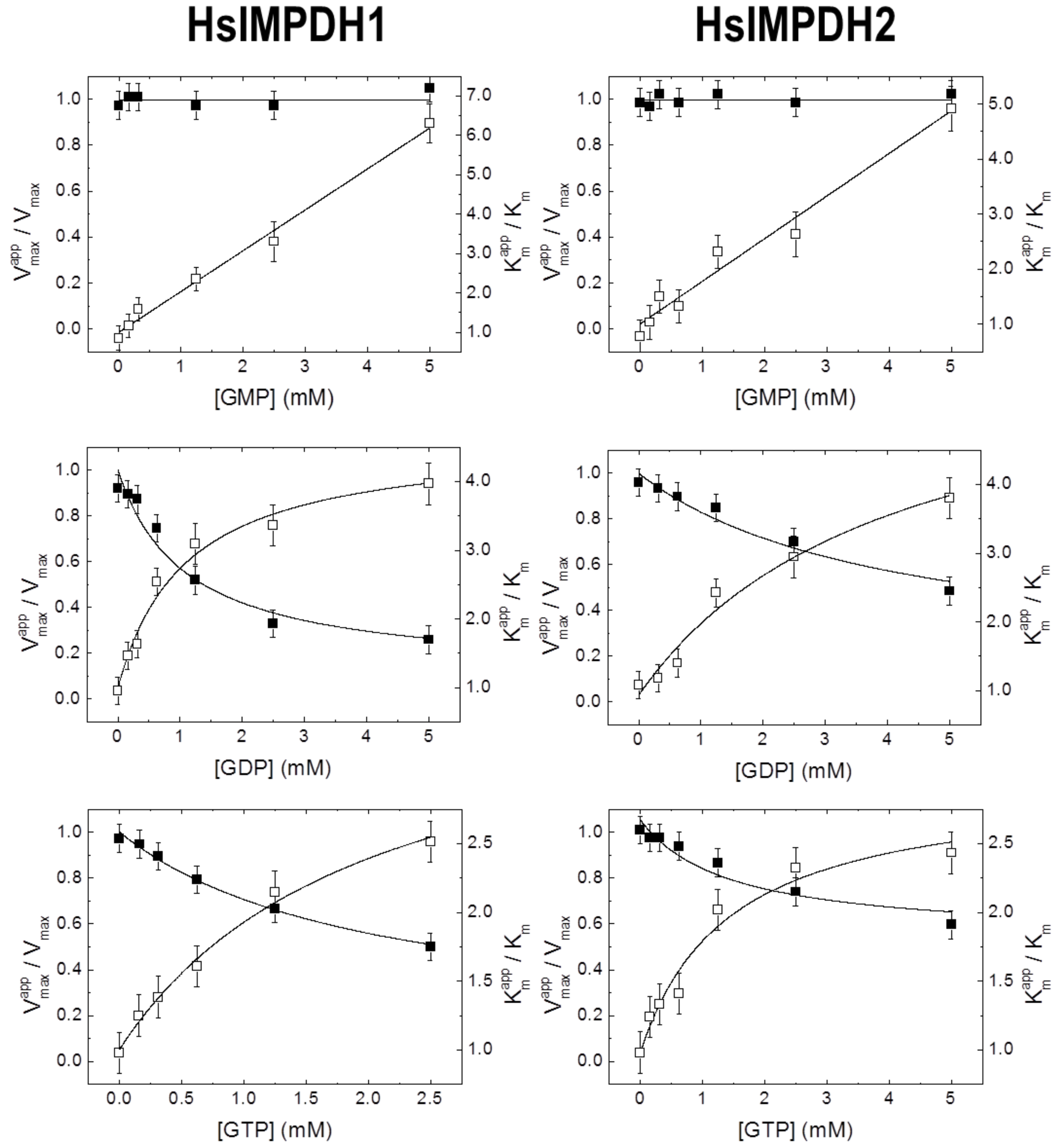

Supplementary Figure 14. *Inhibition of the catalytic activity of HsIMPDHs by guanine nucleotides*. The plots represent the values of V_{max}^{app} / V_{max} (black squares) and K_M^{app} / K_M (white squares) at different concentrations of GMP (upper panels), GDP (middle panels) and GTP (lower panels) for the two human isoforms. The V_{max}^{app}, V_{max}, K_M^{app} and K_M values were obtained by fitting the initial velocities calculated from the time-course enzyme kinetic data to the Michaelis-Menten equation. The error bars represent the standard errors.

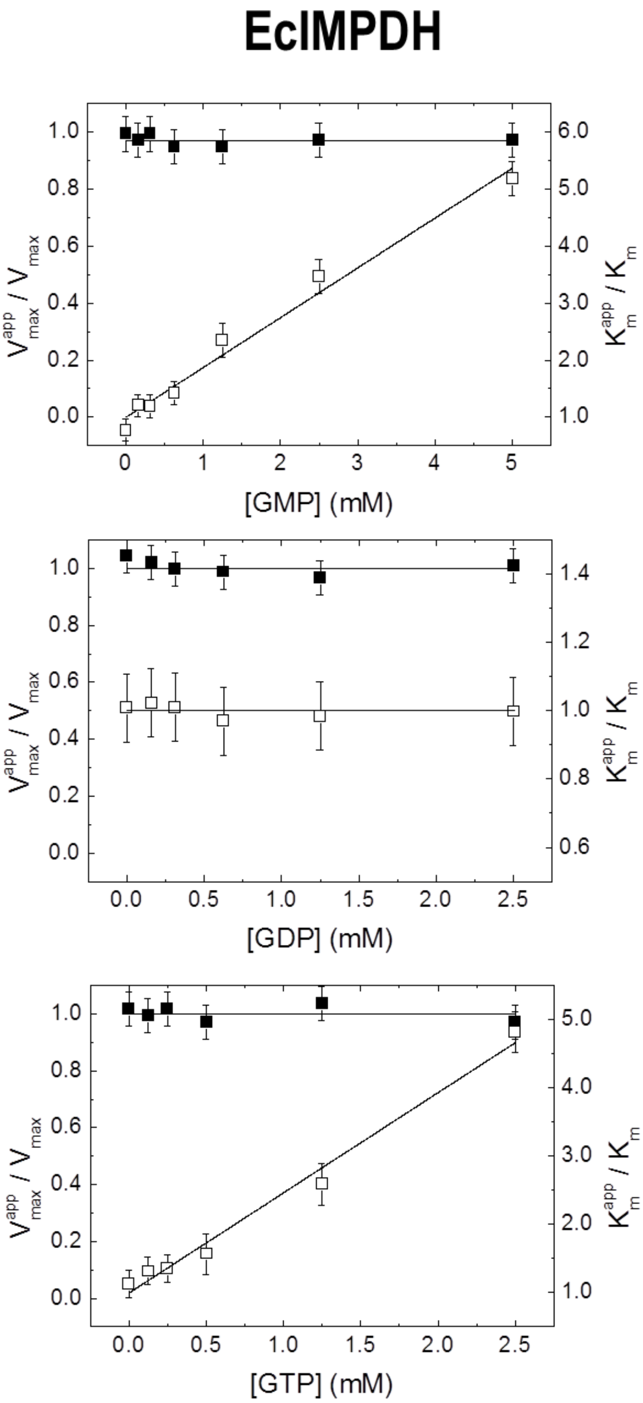

Supplementary Figure 15. *Inhibition of the catalytic activity of EcIMPDH by guanine nucleotides*. The plots represent the values of V_{max}^{app} / V_{max} (black squares) and K_M^{app} / K_M (white squares) at different concentrations of GMP (upper panel), GDP (middle panel) and GTP (lower panel). The V_{max}^{app}, V_{max}, K_M^{app} and K_M values were obtained by fitting the initial velocities calculated from the time-course enzyme kinetic data to the Michaelis-Menten equation. The error bars represent the standard errors.

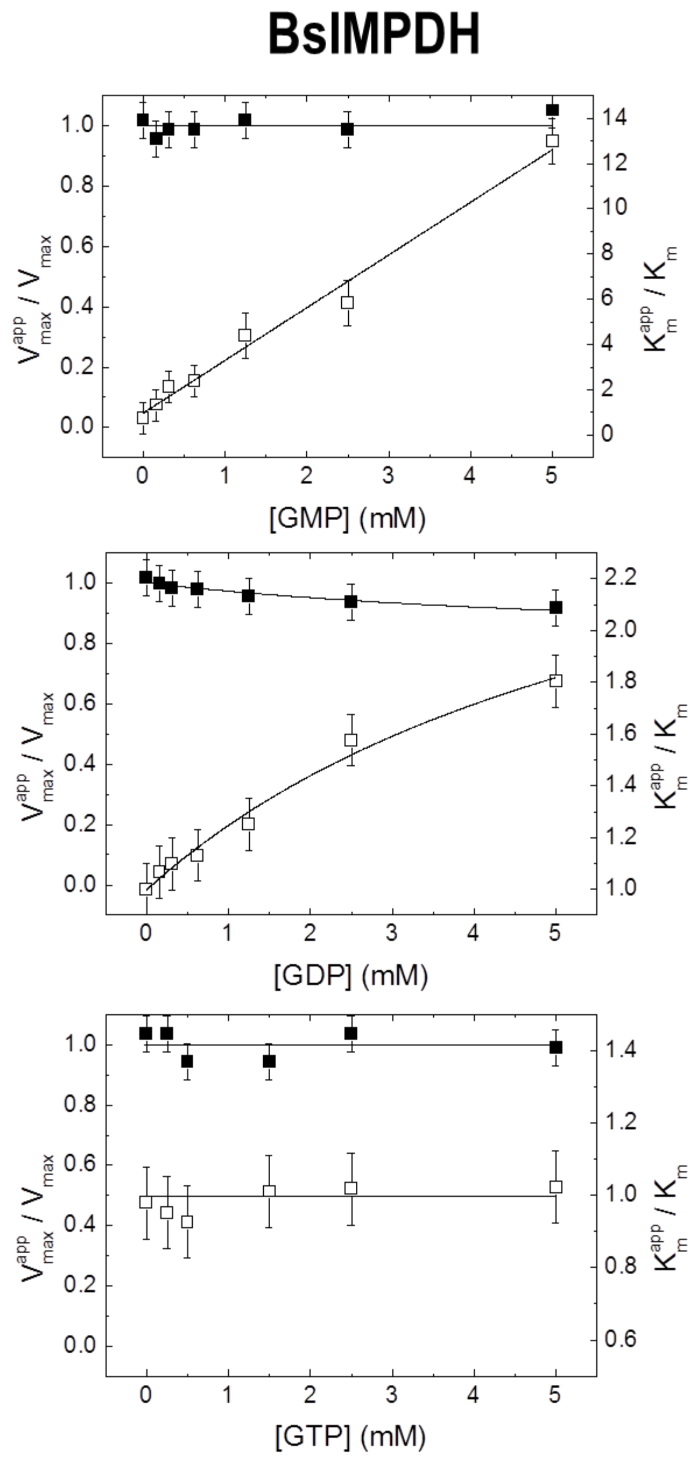

Supplementary Figure 16. *Inhibition of the catalytic activity of BsIMPDH by guanine nucleotides*. The plots represent the values of V_{max}^{app} / V_{max} (black squares) and K_M^{app} / K_M (white squares) at different concentrations of GMP (upper panel), GDP (middle panel) and GTP (lower panel). The V_{max}^{app}, V_{max}, K_M^{app} and K_M values were obtained by fitting the initial velocities calculated from the time-course enzyme kinetic data to the Michaelis-Menten equation. The error bars represent the standard errors.

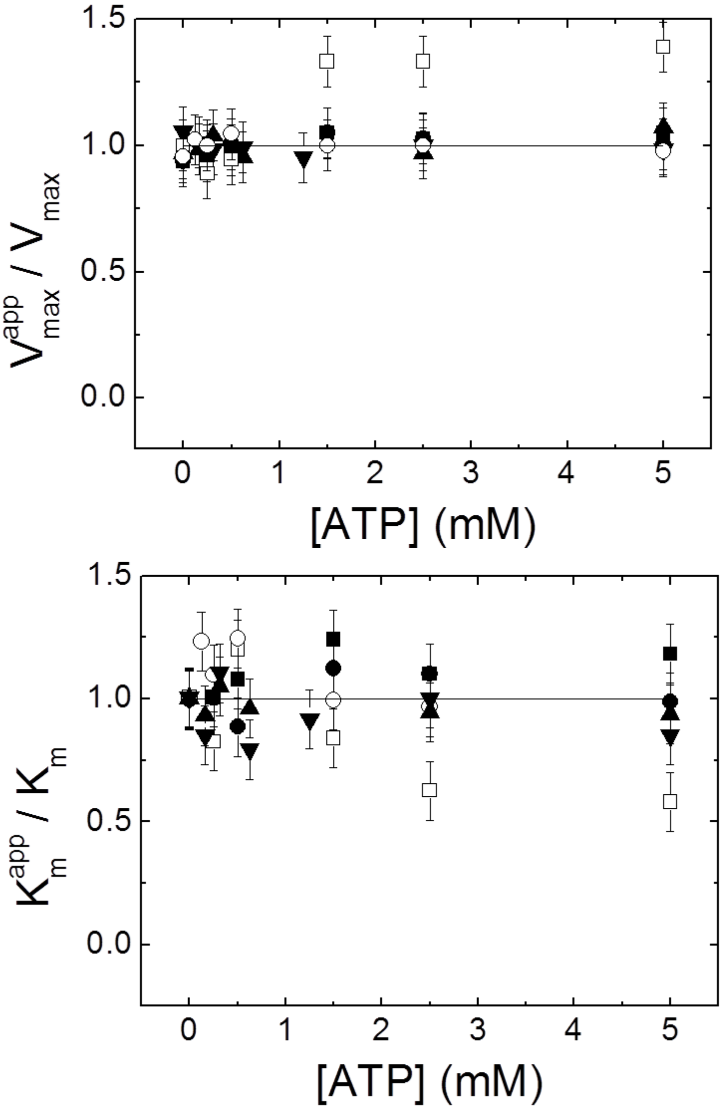

Supplementary Figure 17. *Effect of MgATP on the catalytic activity of IMPDHs*. The plots represent the values of V_{max}^{app} / V_{max} (upper panel) and K_M^{app} / K_M (lower panel) at the indicated concentrations of ATP. Total $MgCl_2$ concentration was 5 mM for all samples except for "AgIMPDH without Mg" (black circles), that contained ATP but no $MgCl_2$. AgIMPDH (black squares), HsIMPDH1 (triangles), HsIMPDH2 (inverted triangles), BsIMPDH (white squares), and EcIMPDH (white circles). The V_{max}^{app}, V_{max}, K_M^{app} and K_M values were obtained by fitting the initial velocities calculated from the time-course enzyme kinetic data to the Michaelis-Menten equation. The error bars represent the standard errors.

A

B

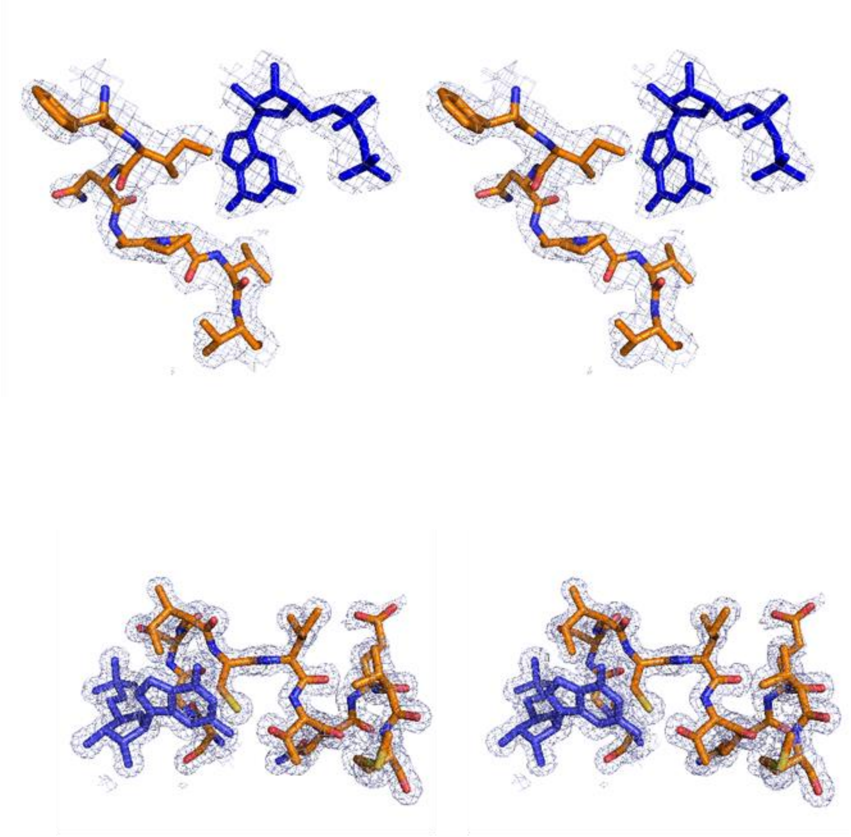

Supplementary Figure 18. *Stereo images of different portions of the electron density map.* **A.** Electron density map (light blue mesh) of residues 120-126 (orange sticks) and GDP2 molecule (blue sticks), extracted from chain B of AgIMPDH-GDP. **B.** Electron density map (light blue mesh) of a portion of the catalytic loop (residues 330-340, shown in orange sticks) as well as the GMP molecule (blue sticks) bound to the active site, extracted from chain A of AgIMPDH-ΔBateman-GMP. The mesh represents the $2mF_o$-DF_c electron density map contoured at 1σ level.

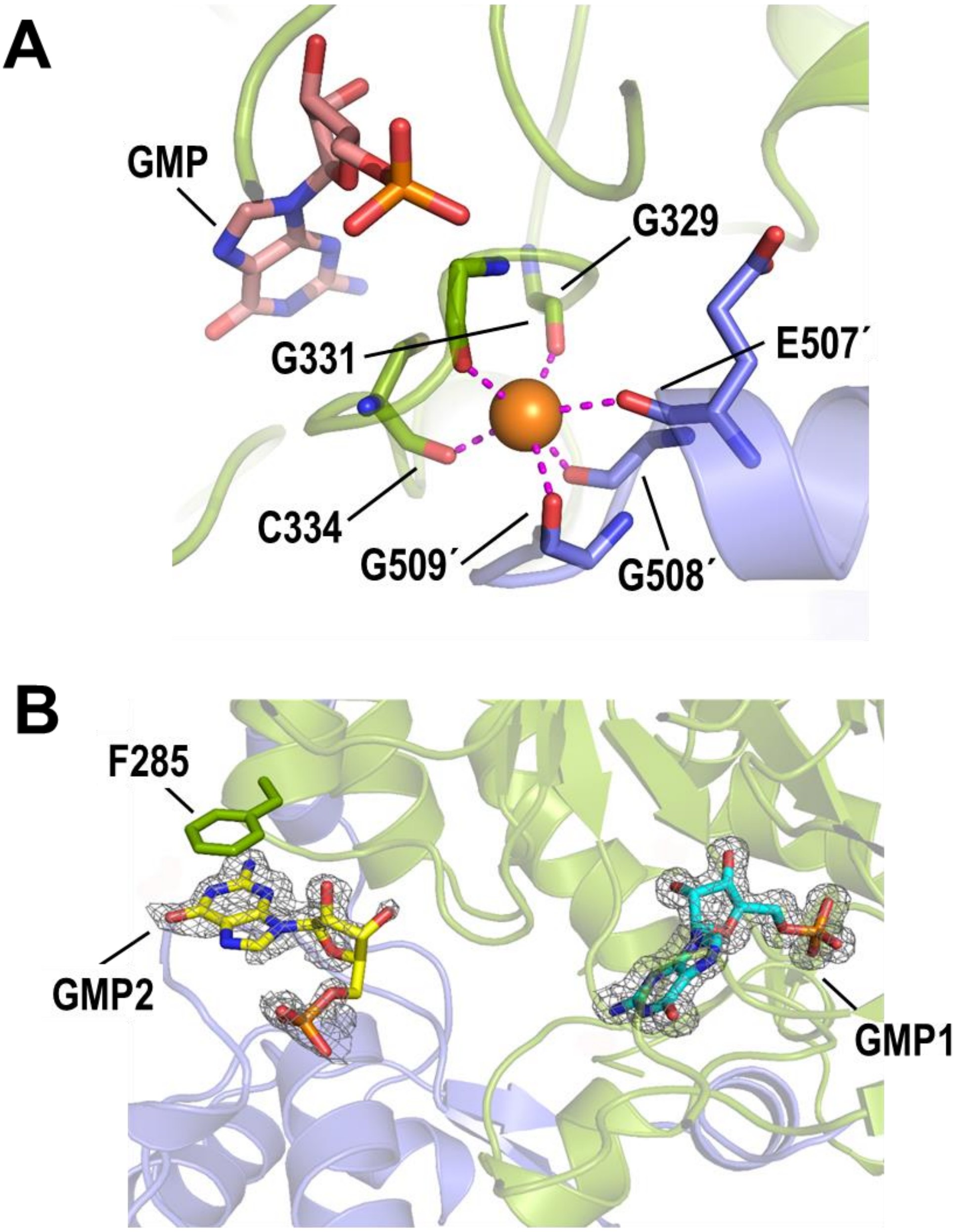

Supplementary Figure 19. *Structure of ΔBateman bound to GMP.* **A**. Close-up view of the K^+ ion bound close to the active site. Protein residues from two adjacent monomers are represented as green and blue cartoons. The GMP bound to the active site is shown as pink sticks and the K^+ atom is shown as an orange sphere. The residues that coordinate K^+ are also shown in green and blue sticks. **B**. View of the two GMP molecules bound per monomer of AgIMPDH. GMP1, bound to the active site, is shown in cyan sticks, while GMP2, unspecifically bound to the adenine sub-site of NAD^+, is represented in yellow sticks. Protein residues from two adjacent monomers are represented as green and blue cartoons. The grey mesh around GMP represents the simulated annealing omit $2mF_o$-DF_c electron density map contoured at 1σ level for both GMP molecules.

SUPPLEMENTARY TABLE

Supplementary Table 1. *ITC-derived thermodynamic parameters.*

	K_d (μM)	ΔH (kcal mol^{-1})	n
**WT**	24	-9.1	0.9
**ΔBateman**	22	-7.7	1.0

GMP titration of AgIMPDH and AgIMPDH-ΔBateman. Estimated relative error in K_d is 15-20%, absolute error for ΔH is 0.3-0.5 kcal mol^{-1} and absolute error for n is 0.05.

SUPPLEMENTARY NOTES

Supplementary Note 1. GMP adopts an identical conformation than IMP into the active site

Crystals of ΔBateman in the presence of 10 mM GMP diffracted up to 1.25 Å resolution (Table 3 in the main text). Two monomers were present in the asymmetric unit (AU), each of them assembled around a quaternary axis that generates a tetramer (Supplementary Fig. 4A). The tetramer is perfectly compatible with our SAXS data (Supplementary Fig. 7A), demonstrating that the crystal structure represents the arrangement found in solution. The slight compaction of the structure (the radius of gyration decreases ~1Å- upon GMP binding (Table 2 in the main text) with respect to apo-AgIMPDH) might be explained by local changes around the active site, possibly in the mobile flap and/or the finger domains that are apparently disordered in apo-AgIMPDH (PDB code 4XWU[1]). In our structure, the catalytic Cys334 loop and the C-terminal segment adopt a transition state-like structure (E-XMP*[2]), with the mobile catalytic flap exposed to the solvent without interpretable electron density for residues 427-444.

GMP is clearly identified bound into the active site (Supplementary Fig. 4B) in essentially identical conformation than the substrate IMP and the product XMP (PDB codes 4XTI and 4XTD[1]). The guanine ring system is stabilized by hydrogen bonds with the backbone of residues Met417, Gly418 and Gly449, as well as the side chains of Thr336. The catalytic Cys334 is at ~2.8 Å distance from the N2 atom of the guanosine ring adopting a catalytically active conformation. Ribose is coordinated to the side chains of residues Ser74, Arg325 and Asp367, while the phosphate moiety binds to the backbone atoms of residues Ser332, Gly369, Gly390 and Gly391,

as well as to the side chain of residues Ser332 and Tyr414 (Supplementary Fig. 4B). Additionally, a K^+ ion is coordinated by the oxygen backbone atoms of residues Gly329, Gly331 and Cys334 in one monomer, and Glu507, Gly508 and Gly509 in the C-terminal part of the adjacent monomer (Supplementary Fig. 19A). K^+ presumably facilitates the conformational changes adopted by the catalytic loop and C-terminal segment along the catalytic cycle[3].

Electron density appears also for a second GMP molecule whose guanine ring occupies the adenine sub-site of NAD^+ (Supplementary Fig. 19B). Nonetheless, the binding of GMP to this site is most probably a crystallographic artifact because ITC experiments unambiguously indicate that AgIMPDH binds one GMP per monomer (Supplementary Table 1) and enzyme kinetics data show that GMP inhibition is purely competitive (Fig. 1A in the main text and Supplementary Fig. 1).

SUPPLEMENTARY REFERENCES

1. Buey RM, Ledesma-Amaro R, Balsera M, de Pereda JM, Revuelta JL. Increased riboflavin production by manipulation of inosine 5'-monophosphate dehydrogenase in *Ashbya gossypii*. *Applied microbiology and biotechnology*, doi: 10.1007/s00253-00015-06710-00252 (2015).

2. Gan L, Seyedsayamdost M, Shuto S, Matsuda A, Petsko G, Hedstrom L. The immunosuppressive agent mizoribine monophosphate forms a transition state analogue complex with inosine monophosphate dehydrogenase. *Biochemistry* **42**, 857-863 (2003).

3. Riera TV, Zheng L, Josephine HR, Min D, Yang W, Hedstrom L. Allosteric activation via kinetic control: potassium accelerates a conformational change in IMP dehydrogenase. *Biochemistry* **50**, 8508-8518 (2011).
